# Supplementary material for: Mitochondria dysregulation contributes to secondary neurodegeneration progression post-contusion injury in human 3D in vitro triculture brain tissue model
Source: Cell Death Dis. 2023 Aug 3;14(8):496. doi: 10.1038/s41419-023-05980-0 (PMC10400598; doi:10.1038/s41419-023-05980-0)
Supplement: Supplementary file 1 — Supplementary Material [file 41419_2023_5980_MOESM1_ESM.docx]

**Supplementary document**

**Mitochondria dysregulation contributes to secondary neurodegeneration progression post-contusion injury in human 3D in vitro triculture brain tissue model.**

Volha Liaudanskaya^1^, Nicholas J Fiore^1^, Yang Zhang^1^, Yuka Milton^1^, Marilyn F Kelly^1^, Marly Coe^1^, Ariana Barreiro^1^, Victoria K Rose^1^, Matthew R Shapiro^1^, Adam S Mullis^1^, Anna Shevzov-Zebrun^1^, Mathew Blurton-Jones^2^, Michael J Whalen^3^, Aviva J Symes^4^, Irene Georgakoudi^1^, Thomas JF Nieland^1^, David L Kaplan^1^

^1^Department of Biomedical Engineering, Tufts University, Medford, MA, USA; ^2^Department of Neurobiology and Behavior, University of California Irvine, CA, USA; ^3^Department of Pediatrics, Massachusetts General Hospital, Charlestown, MA, USA; ^4^Department of Pharmacology and Molecular Therapeutics, Uniformed Services University, MD, USA

**Supplementary information**

**For Figure 6. Mitochondria dysregulation-induced neurodegeneration and inflammation are independent of the microglia cell source.**

After 6 weeks, the 3D tricultures went through the injury protocol, and the effect of contusion injury was observed after 24 hours (Fig. 6; Supplementary Fig. 16-17). The neuronal network degraded by 50% (p<0.05) in cultures containing microglia cells from either donor, and the effect was reversed in the presence of P110 (Fig. 6 a-c; Supplementary Fig. 23 a, b). The degradation of the neuronal network was associated with increased mitochondria fission marker expression and pDRP1 (both lines); however, we did not observe an increase in Fis1 expression in either of the lines (Fig. 6 d; Supplementary Fig. 23 c). In NAMc made with either of the iPSC microglia lines, the expression of pDRP1 was reversed in the presence of P110. Unlike in tricultures with the HMC3 cells, levels of Mitofusin 1,2 protein were downregulated in the iPSC tricultures after injury, while the treatment with P110 peptide was not significant from either sham or injury group without treatment (Supplementary Fig. 24), suggesting decreased fusion. Similar to HMC3 tricultures (Fig. 4-5), we observed mitochondria fission-induced neuroinflammation; A1 astrocyte-associated cytokines IL-1α, IL-1β, and TNFα were upregulated in untreated injured cultures and downregulated to the levels of a sham when treated with P110 (Fig. 6 e, Supplementary Fig. 24, 25). Increased levels of ECM remodeling markers MMP2 and MMP3 were reduced by P110 in injured HMC3 but not in YZ1 or ND41866*C tricultures (Fig. 6e, Supplementary Fig. 26). mRNA sequencing cluster analysis revealed significant changes in ECM assembly and function, further suggesting ECM contribution in injury progression (Supplementary Fig. 27-28). The apoptosis marker FasL expression was similarly upregulated in all microglial tricultures; however, the levels were reduced by P110 treatment only in HMC3 tricultures. mRNA sequencing cluster analysis revealed enrichment of type I interferon response and major histocompatibility complex (MHC) class I antigen presentation in response to contusion injury (Fig. 6 f, g – cluster 6, Supplementary Fig. 27, 28), which correlates with genetic signature observed in rodent studies of brain injuries (1). We next looked at the expression of glia-specific gene-contributors to interferon type I response activation, known to be dysregulated post fluid percussion injury model in rodents (25 top DEGs), and detected similar changes in our contusion human *in vitro* injury model (Upregulated - SLFN5, OAS3, PARP12, TRIM25, CCL2, PARP9, ISG15, PAPR14, IFIT2, 3, IFI16, OAS1, SP100, GDP2, EIF2AK2, IFIH1, ATP10A, MLH1; downregulated - MTHFR, C3, XDH, IRGM, SDC3, NAMPT) (Supplementary Fig. 29). We have found greater transcriptional activation of microglia specific markers, compared to astrocytes, which has been shown *in vivo* rodent injury studies as well (1). Some of the markers were not detected in our model (some examples are IFI209, OASL2, IFI27I2A, IRF9, and H2-Q4), due to species differences and limitations of our model (lack of macrophages, blood vessels, and other factors) (1). Among upregulated genes in astrocytes were GBP3, IFIT3, and downregulated MAFB. Microglia genes upregulated were PARP14, PHF11, IFIT3, IFI16, MF213, MLH1, SRP54, MYO1E, and downregulated SDC3 (Supplementary Fig. 29).

At last, we have discovered that astrocytes-specific pan-markers were significantly downregulated, together with neuron-specific markers (Fig. 6 f-g, cluster M1, Supplementary Fig. 27, 30-31), while microglia markers were mostly unchanged or upregulated (Supplementary Fig. 30). Indicating, the potential death of neurons and astrocytes, but not microglia. Additionally, cluster analysis of cell cycle gene expression revealed activation of the cell cycle and DNA repair progression (Fig. 6 f, g – cluster 5).

**For Figure 7. Contusion injury induced mitochondria intra- and extracellular changes in human 3D triculture model of brain injury.**

To confirm microglial mitochondria fission contribution to neurodegeneration, the transfer experiments were replicated in iPSC microglia-based models (Fig. 7 d-k; Supplementary Fig. 23 e, f, 33). Structural damage and mitochondrial fragmentation were observed in neurons that received extracellular mitochondria from microglial exposed to conditioned media isolated from injured neurons (50% of the network degraded in both iPSC lines; N-M-Nt). P110 treatment counteracted these effects (Supplementary Fig. 33). Extracellular mitochondria isolated from astrocytes (after they were treated for 24 hours with microglial mitochondria activated with conditioned media from injured neurons, please see Fig. 5a for experimental design) exacerbated neurodegeneration in transfer experiments with the ND41866*C line to 75% of network degradation (N-M-A-Nt; Supplementary Fig. 23 e, f) and remained similar in YZ1 cultures (50% of network degradation) (Fig. 7 d-g) These effects were associated with increased levels of mitochondria fragmentation in both lines (Fig. 7 f, g; Supplementary Fig. 23 e, f).

We examined the metabolic profile of mitochondria released from neurons, astrocytes, and iPSC microglia (YZ1 line) after exposure to conditioned media from injured neurons (Fig. 7 h-k; N-M-A-Nt). Similar to transfer experiments with the HMC3 line, we observed an increased level of redox ratio in microglial mitochondria following the transfer of conditioned media from injured neuronal cultures. P110 treatment mitigated this increase. Mitochondria isolated from astrocytes that had been activated with microglial mitochondria showed an increased redox ratio for the P110 injury group. No change was detected after the last transfer step to neurons (Fig. 7 i). The NADH bound fraction was consistently lower in P110-treated astrocytes and neuronal groups, with no changes in the microglia group (Fig. 7 j). However, the astrocytes control injury group showed an increase in NAD(P)H bound fraction, and no change was observed in the neuronal control group, unlike in HMC3 experiments. The long lifetime measurements were similar between the HMC3 and the YZ1 line. Together these findings confirmed cell-line independent effects of microglial mitochondria fission on secondary neurodegeneration and neuroinflammation progression after contusion injury.

**Supplementary Results**

Initially, cultures were incubated in a Neurobasal medium supplemented with 1% Anti-Anti, 1% Glutamax, and 2% B27 supplement, and cellular viability was checked weekly with calcein (live) staining. Two weeks after seeding, astrocytes in monocultures and co-cultures started to die rapidly (Figure Supplementary 1a, red arrows indicate dying cells). To prevent astrocytic death, 1% astrocytes growth factors (ScienCell Research Laboratories) were introduced in the previously described neuronal medium, and viability was checked weekly. Astrocytes in monocultures showed rapid growth and proliferation. In co-cultures and tricultures, we observed high neuronal network density formation without the obvious presence of dead cells (Supplementary Fig. 1 b). For all consequent experiments in this manuscript, cultures were incubated in the Neurobasal medium supplemented with astrocytes growth factors, B27, Glutamax, and anti-anti.


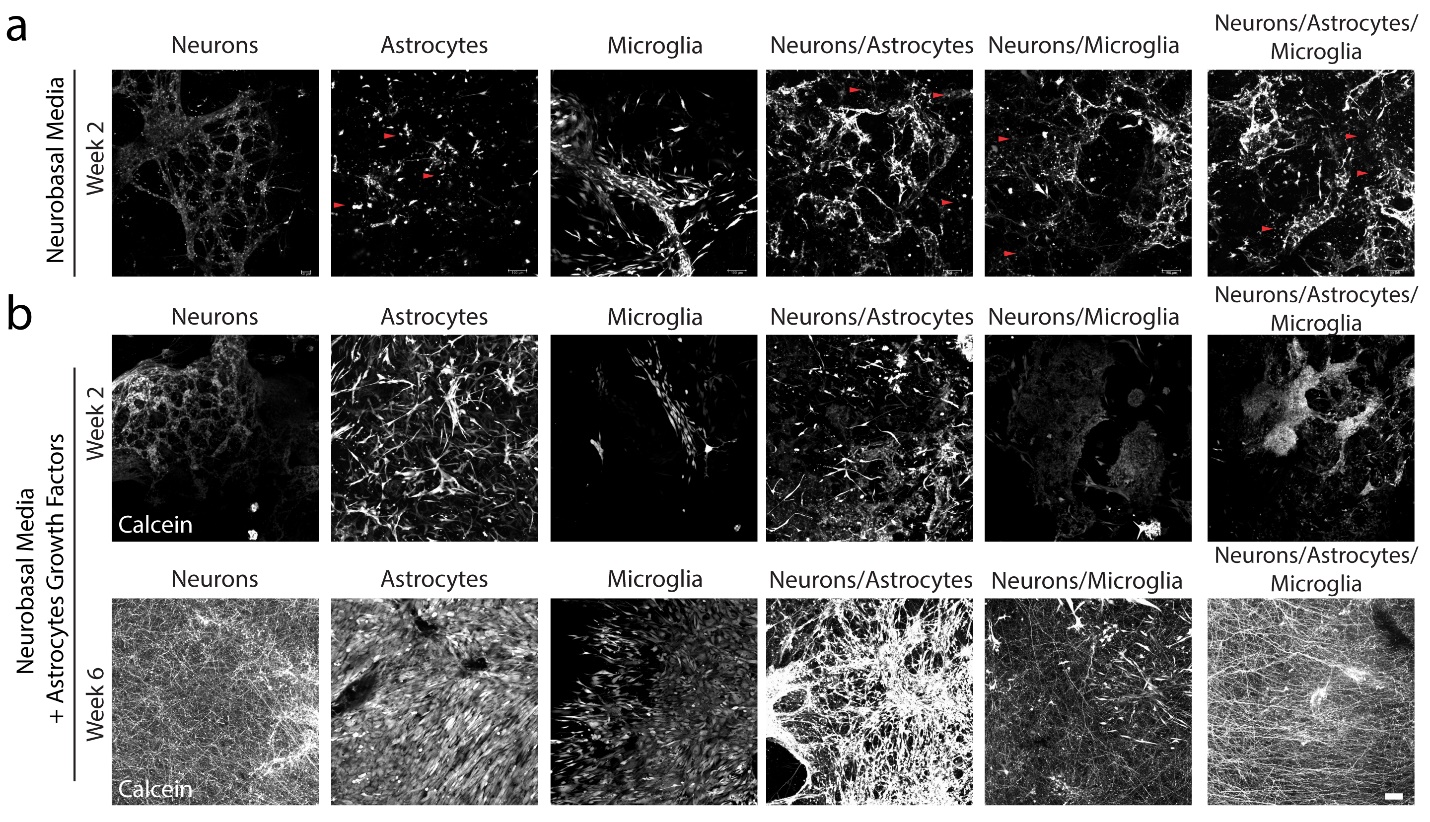


**Supplementary Fig. 1. Astrocyte growth factors were crucial in maintaining the viability of 3D tissues over time.** **a,** Representative images of calcein-stained live samples at 2 after seeding. Astrocytes and co-cultures with astrocytes demonstrated a high death rate when incubated in a neurobasal medium without astrocytes growth factors 2 weeks after seeding. Red arrows point to dead cells. **b,** Addition of astrocytes growth factors into the Neurobasal medium supported all cultures' growth and development at 2 and 6 weeks after seeding. Scale bar: 100μm.


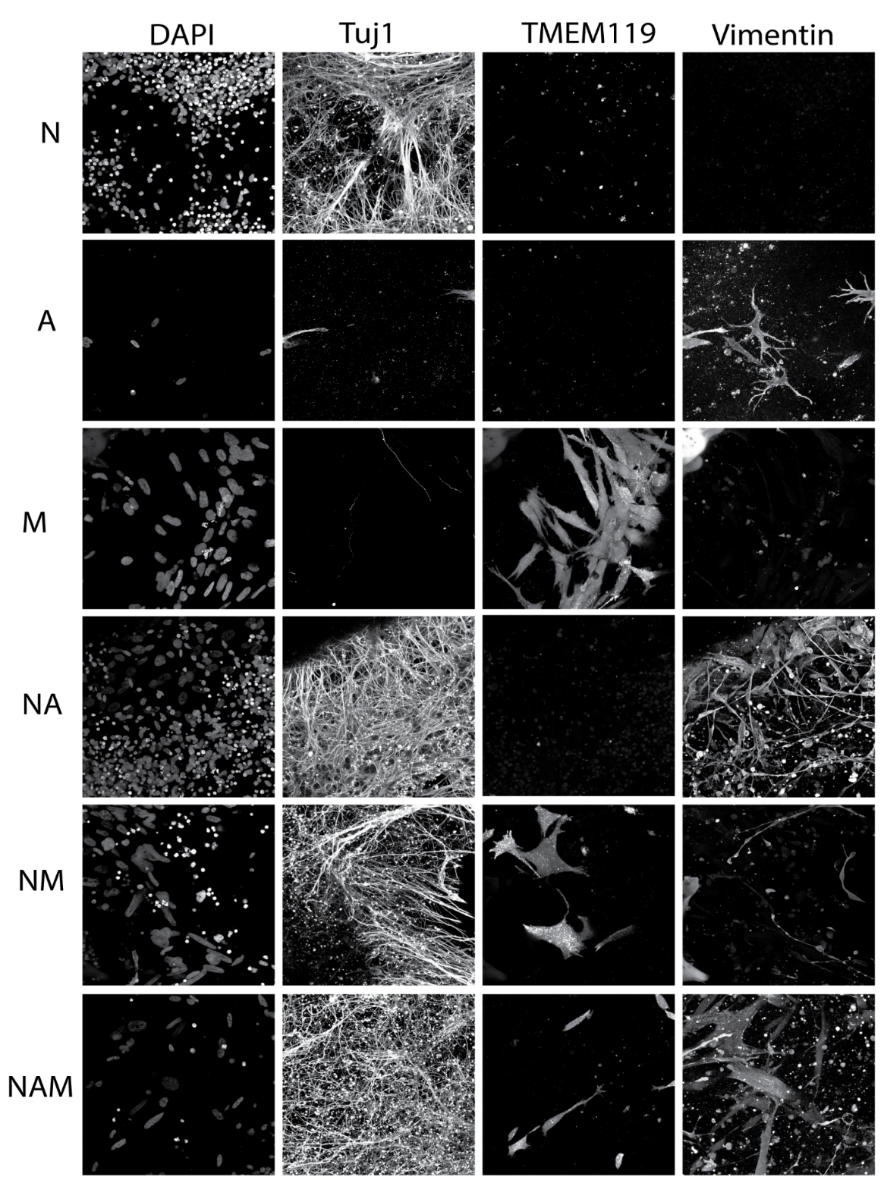


**Supplementary Fig. 2 Human in vitro mono-, di- and triculture model composed of neurons, microglia, and astrocytes.**  Representative images of neuronal (Tuj1), microglia (TMEM119), and astrocytes (vimentin) specific cytoplasmic markers in mono-, di- and tricultures. Scale bar: 50μm.


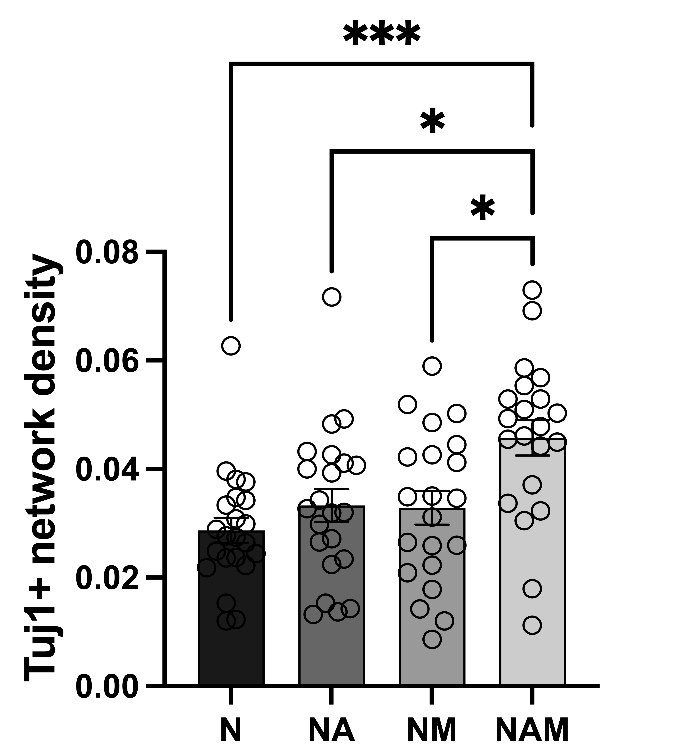


**Supplementary Fig. 3 Human in vitro mono-, di- and triculture model composed of neurons, microglia, and astrocytes.** Quantification of Tuj1 positive network from Supplementary Fig. 2. Datta presented as mean ±SEM of three independent experiments with n=4 scaffolds per condition. *, *** indicates significant differences (p<0.05, 0.001 respectively; two-way ANOVA (analysis of variance) (with Tukey’s post-hoc tets) between control and experimental groups).

Gene expression analysis in tricultures demonstrated a high level of neuronal quintessential (TUBB3, PAX6, SYN1) and maturation markers (MAP2, RBFOX3), cortical layer-specific genes (RELN, CUX1, POU3F2, BCL11B, FOXP2), excitatory and inhibitory neuronal markers (ATP12A2, TH, SLC6A1, ACHE, GRIN2B, ISL2) (Supplementary Fig. 4a); high levels of astrocytes (SOX9, AQP4, GFAP, S100B, ALDOC, SLC1A2, ALDH1A1, ALDH1L1) and microglia pan markers (HEXB, IRF8, RUNX1, SPI1, TAL1); and low to undetectable levels of pluripotency (OCT4, NANOG) or brain endothelial cells markers (VWF, CDH5M, CD34) (Supplementary Fig. 4b) (2). Microglia-specific genes were expressed at a lower level due to a small percentage of these cells present in the tricultures (4%) in comparison to neurons (77%) or astrocytes (19%). To further analyze the state of neuronal maturation in the 3D triculture in vitro model, the gene expression was compared to the earlier published genetic footprint of iPSC-derived neurons in monocultures, co-cultures with astrocytes, and adult human neurons (Supplementary Fig. 5 a-d) (2). Gene expression of markers associated with adult neurons and maturation of iPSC-derived neurons (particularly in co-culture with astrocytes) were expressed at comparable levels in the human 3D in vitro triculture model (Supplementary Fig. 5 b-d) (2).


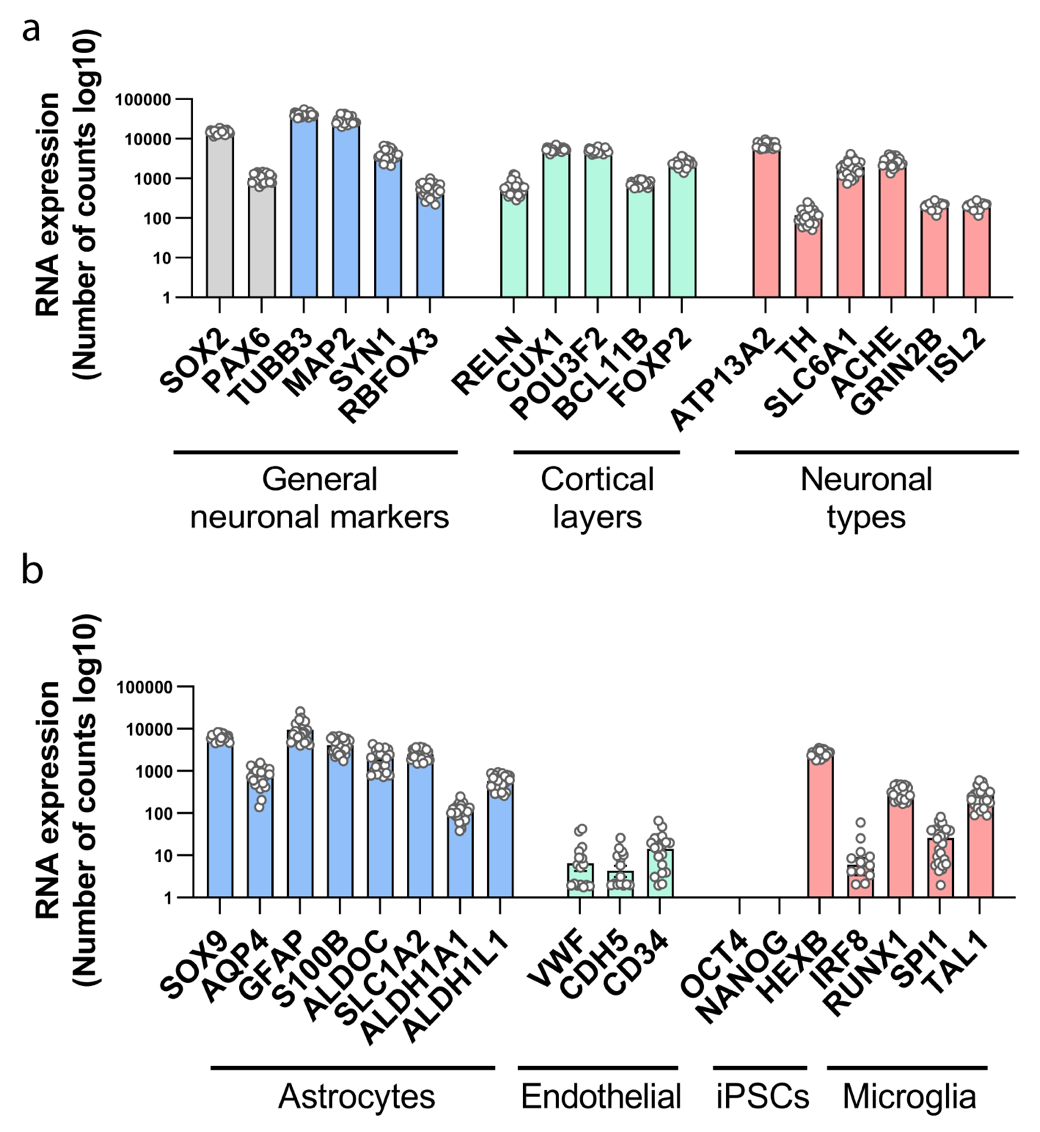


**Supplementary Fig. 4 Human 3D in vitro triculture model characterization.** mRNA expression (count number via bulk mRNA sequencing) of **a,** neuron-specific makers (SOX2, PAX6 - progenitor state; TUBB3 – pan-marker; MAP2 – dendritic; SYN1 – synaptogenesis; NeuN – transcriptional factor of neuronal maturation): cortical layers specific (RELN – Layer I; CUX1 – Layer II-IV; POU3F2 – Layer V-VI; BCL11B – Layer V, striatal neurons; FOXP2 – Layer V-VI); and neuronal subtypes specific (ATP12A2, TH – Dopaminergic neurons; SLC6A1 – GABAnergic; ACHE – Cholinergic; GRIN2B – Glutaminergic; ISL2 – motor neurons)); **b,** astrocyte-specific (SOX9, AQP4, GFAP, S100B, ALDOC, SLC1A2, ALDH1A1, ALDH1L1); endothelial specific (VWF, CDH5, CD34); iPSC (OCT4, NANOG); microglia specific (HEXB, IRF8, RUNX1, SPI1, TAL1) markers. Each marker shows n=12 samples isolated from tricultures composed of induced neural stem cells derived neurons, primary astrocytes, and iPSC-derived microglia from two healthy donors, YZ1 and ND418664*F.


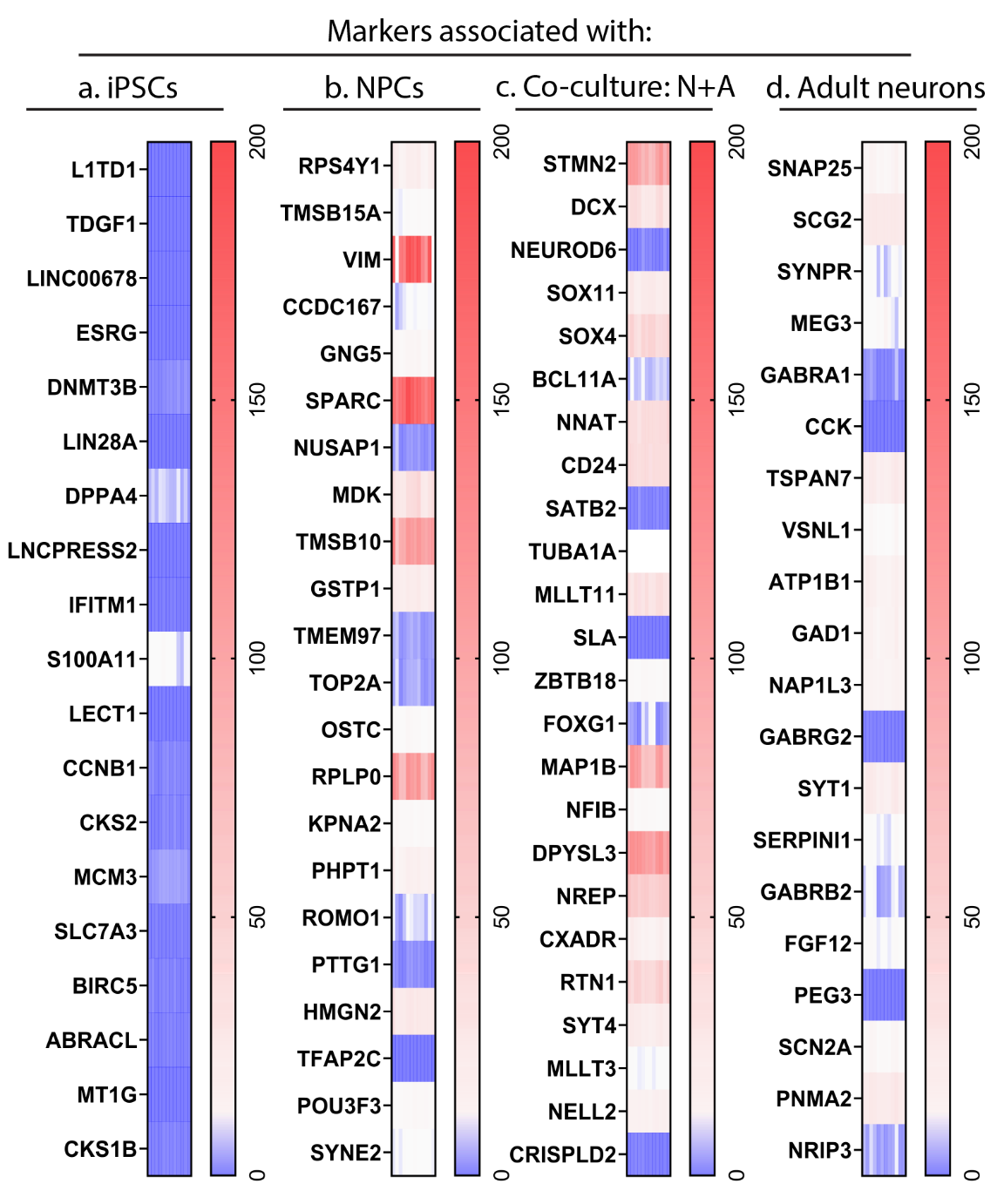


**Supplementary Fig. 5 Neuronal Maturation markers are highly expressed relative to hiPSC markers in the human 3D in vitro triculture model.** mRNA expression (count number via bulk mRNA sequencing) of **a,** induced pluripotent stem cell (iPSCs); **b,** neural progenitor cells (NPCs); **c,** iPSCs-derived neuronal + astrocytes co-culture; and **d,** human adult neuronal markers. Each marker shows n=12 samples isolated from tricultures composed of induced neural stem cells derived neurons, primary astrocytes, and iPSC-derived microglia from two healthy donors, YZ1 and ND418664*F.

To ensure a non-inflamed state of glia in co-culture conditions, the production level of inflammatory cytokines, chemokines, and growth factors was evaluated (Supplementary Fig. 6). Among 24 profiled markers, the level of IL-8 chemokine was significantly higher in co-cultures conditions, comparing to neuronal monocultures, and in triculture, IL-8 was not different from NA or NM di-cultures. Other markers were equal between all groups, indicating a homeostatic and stable environment.


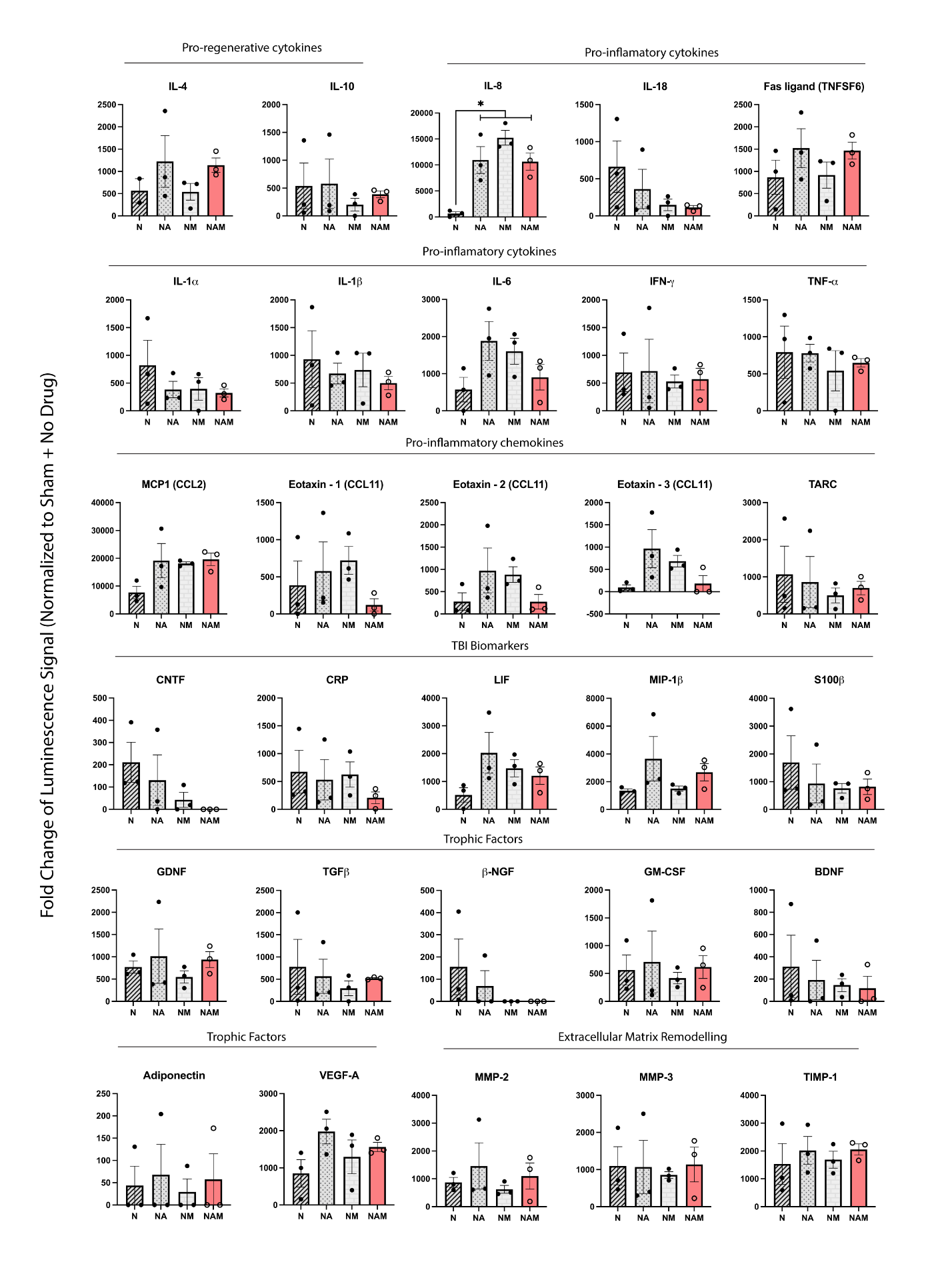


**Supplementary Fig.6.** Neuronal co-culture with astrocytes and/or microglia shows immune homeostasis 6 weeks after seeding. The production level of Pro-regenerative and pro-inflammatory cytokines and chemokines, TBI biomarkers, Trophic factors, and ECM remodeling proteins. Data presented as mean ± SEM of three independent experiments with each data point is the average for n=4 scaffolds per condition. *, **, ***, **** indicate significant differences (p<0.05, 0.01, 0.001, 0.0001, respectively; two-way ANOVA (analysis of variance) between control and experimental groups).

Contusion injury resulted in the release of two brain injury-associated biomarkers, lactate dehydrogenase (3) and glutamate (4), in all mono-, di- and tricultures within 48hr (Supplementary Fig. 7). Only in the triculture conditions did the increased release of LDH and glutamate persisted for a prolonged time (14 days), after 40% drop at earlier time points 24 and 48hr. P110 treatment had no effect on the glutamate and LDH release, which is consistent with other drug treatment protocols, where neuronal protection was not associated with LDH or glutamate concentration (data not shown).


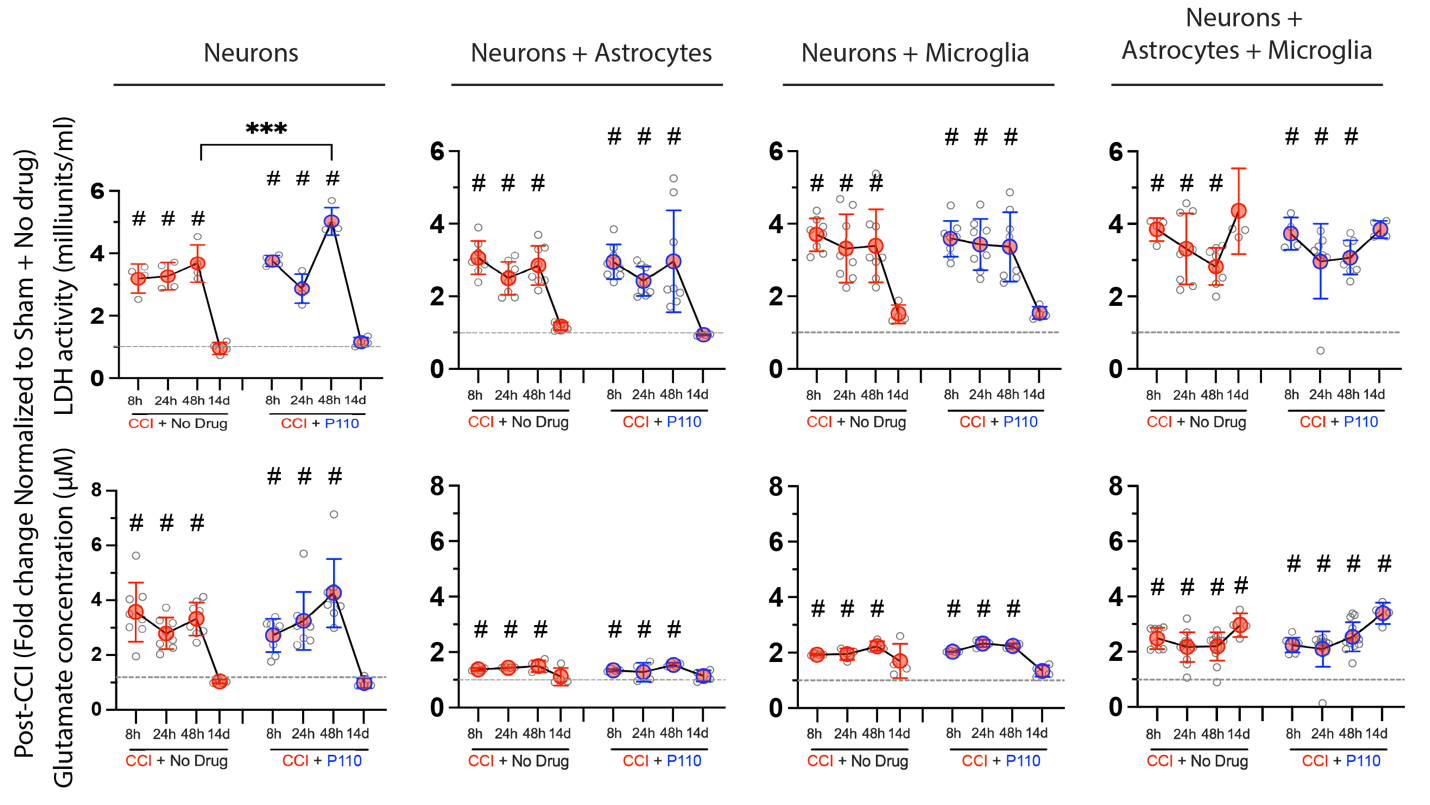


**Supplementary Fig. 7. Biochemical response to TBI in human 3D in vitro model. Top,** LDH and **Bottom,** Glutamate release after controlled cortical impact injury in human 3D cultures of neurons, microglia, and astrocytes in mono-, di- and tricultures at 4-time points: 8, 24, 48 hours, and 14 days. Data presented as mean ±SEM of three independent experiments with n=2-4 scaffolds per condition. *, **, ***, **** indicate significant differences (p<0.05, 0.01, 0.001, 0.0001 respectively; two-way ANOVA (analysis of variance) (with Tukey’s post-hoc test) between control and experimental groups).


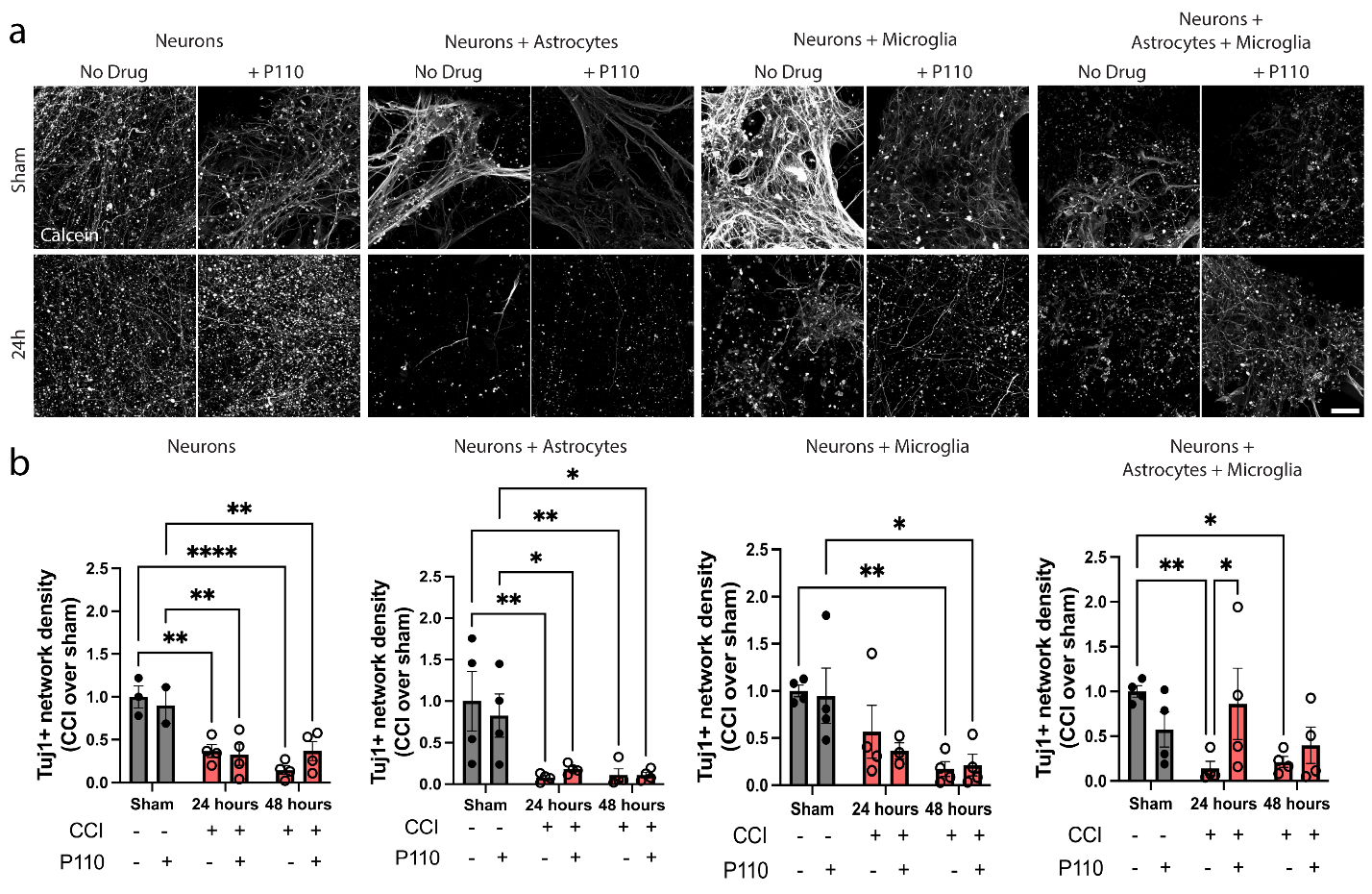


**Supplementary Fig. 8 Neuronal network degeneration in the injury site 24 hours after controlled cortical impact injury.** **a,** Representative images of Tuj1 neuronal staining at 24 hours after CCI in N, NA, NM, and NAM groups. **b,** Quantification of Tuj1 neuronal network density in control and P110 treated groups of N, NA, NM, and NAM at two different time points (24 and 48 hours). Data presented as mean ±SEM of three independent experiments with n=2-4 scaffolds per condition. *, **, ***, **** indicate significant differences (p<0.05, 0.01, 0.001, 0.0001, respectively; two-way ANOVA (analysis of variance) between control and experimental groups). Scale bar: 50 µm.


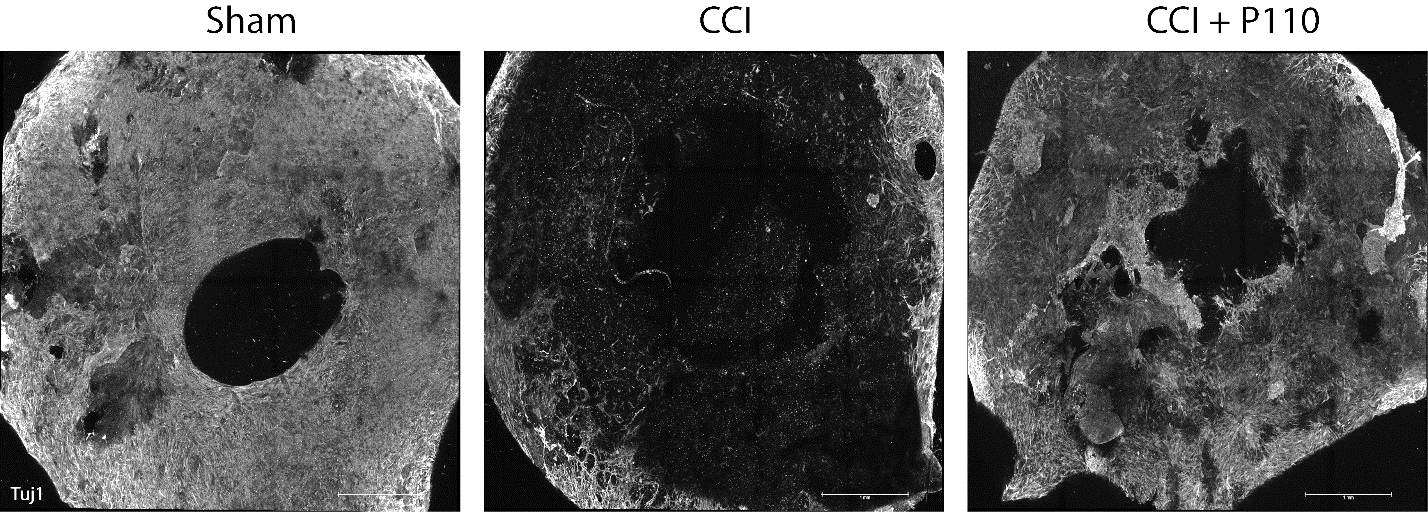


**Supplementary Fig. 9. Contusion injury induces mitochondria fission-dependent progressive neuronal network degradation in the human 3D triculture model.** Representative images of Tuj1 neuronal network staining at 24 hours after the injury in NAM groups with and without P110 treatment. Scale bar: 1mm.


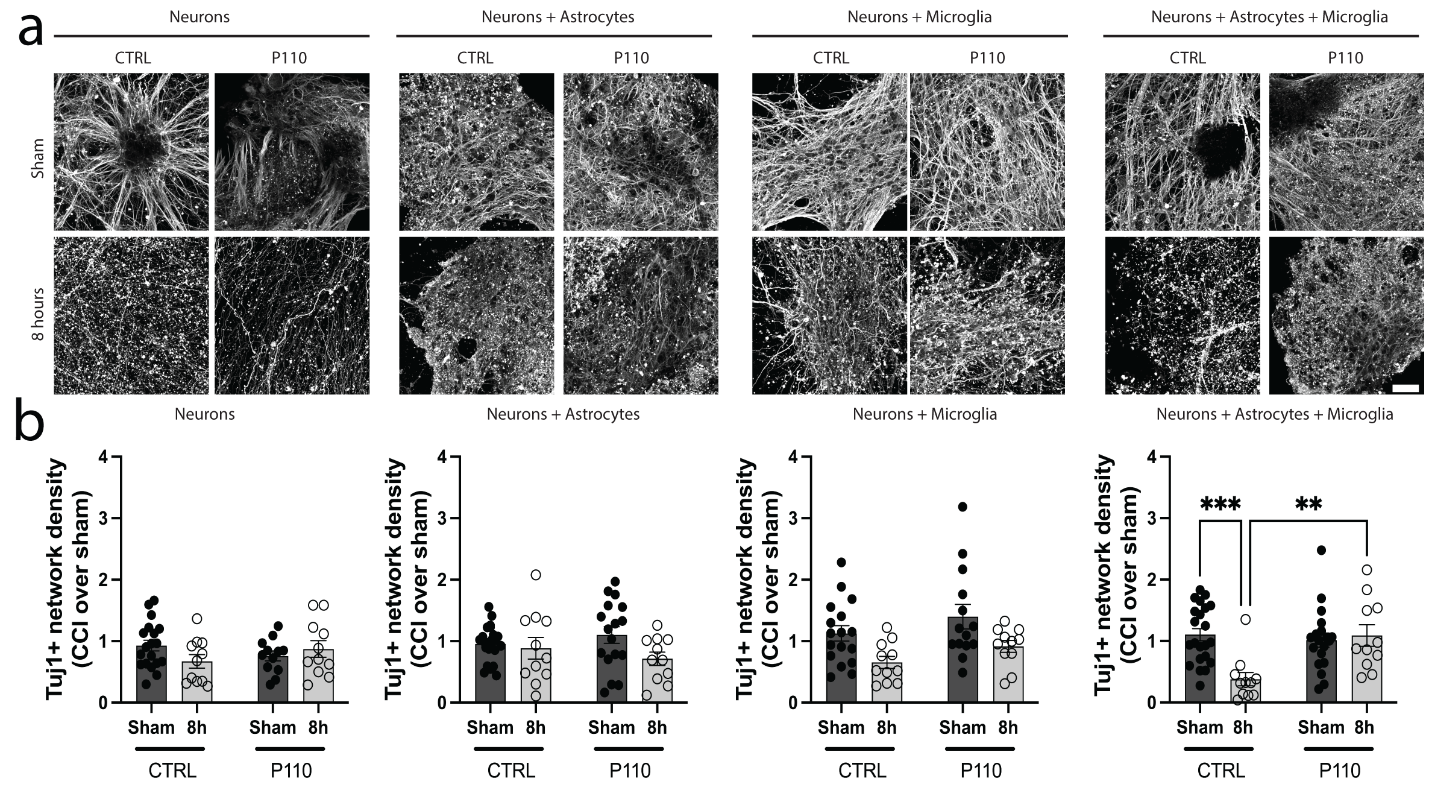


**Supplementary Fig. 10. Crosstalk between astrocytes and microglia had a detrimental effect on neuronal survival after brain injury and was reversible with mitochondria fission inhibitor P110.** **a,** Representative images of Tuj1 neuronal staining at 8 hours after contusion in N, NA, NM, and NAM groups in the proximal area. **b,** Quantification of Tuj1 neuronal network density in control and P110 treated groups. Data presented as mean ±SEM of three independent experiments with n=2-4 scaffolds per condition. *, **, ***, **** indicate significant differences (p<0.05, 0.01, 0.001, 0.0001, respectively; two-way ANOVA (analysis of variance) between control and experimental groups).


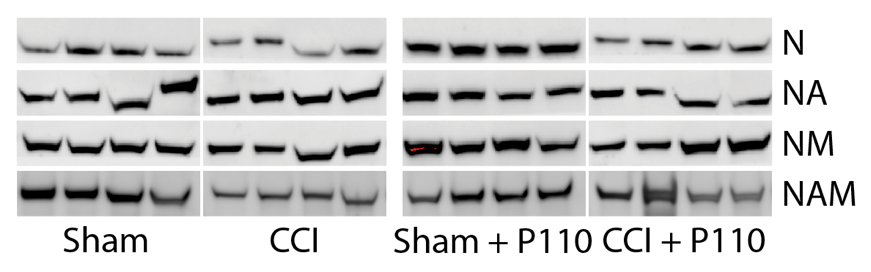


**Supplementary Fig. 11. Injury induces neuronal network degradation in N and NAM groups 24 hours post-impact—**representative western blots of MAP2 marker in all groups. Proteins were isolated from the entire scaffolds without separating the injured area from the penumbra.

Next, we looked at the activation of the necroptotic pathway, a known mechanism of neuronal death post-controlled cortical impact injury in rodents (5). We have confirmed necroptotic death execution in Nc and NAMc (the groups that showed neuronal network degeneration) through phosphorylation of mixed lineage kinase (pMLKL), known as necroptotic executor (Supplementary Fig. 11). While the early marker of necroptotic death – phosphorylation of receptor-interacting serine/threonine-protein kinase 3 (RIP3) has not been detected (Supplementary Fig. 11). At last, P110 peptide had no significant effect on the progression of necroptotic death, indicating that P110 acts downstream of necroptosis activation and progression.


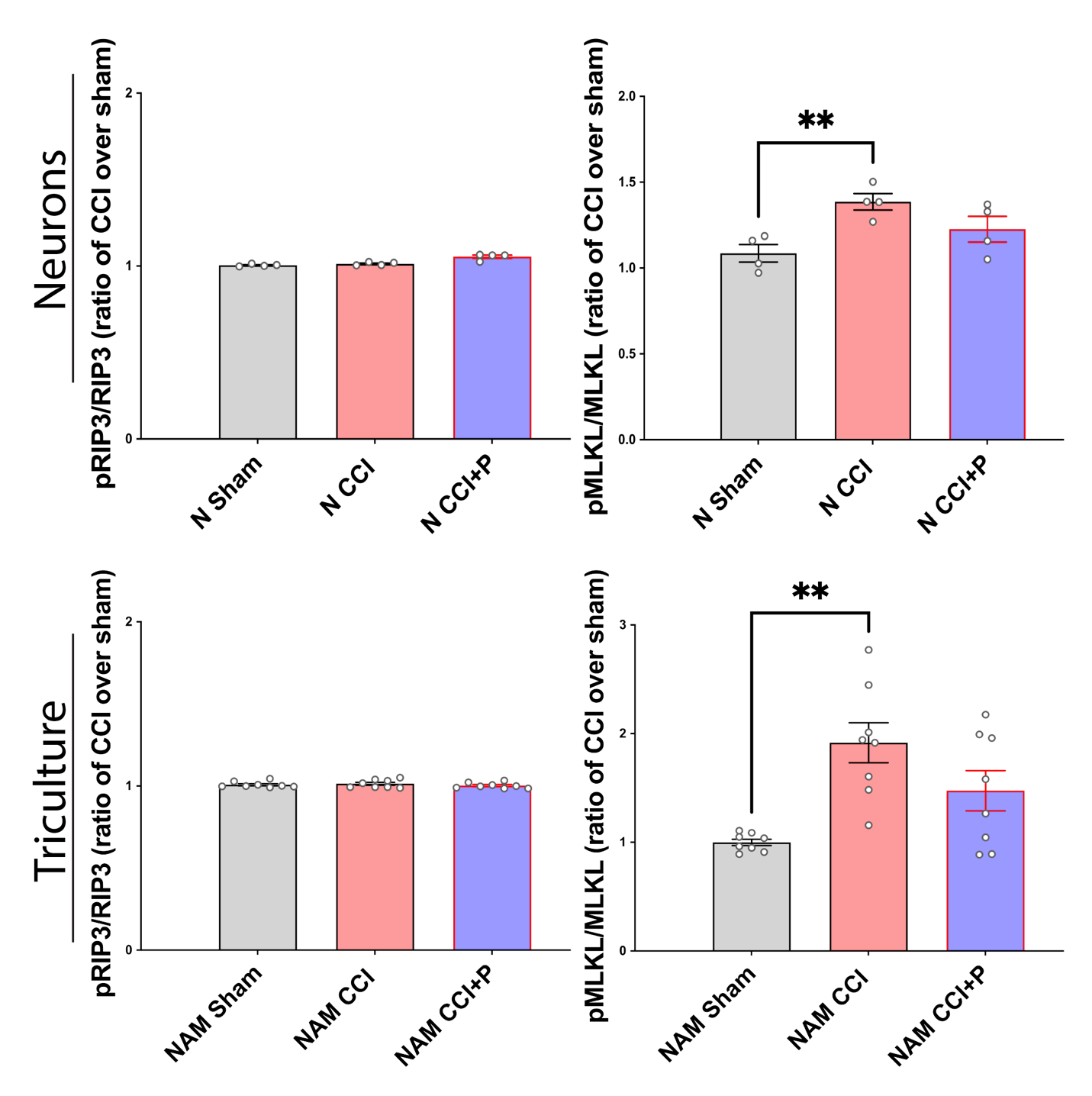


**Supplementary Fig. 12 Contusion injury induces necroptotic death progression independent of mitochondria fission in neuronal monoculture and tricultures.** Densitometry analyses of phospho-RIP3 (p-RIP3) and p-MLKL protein amount 24 hours after contusion injury in control and P110 treated groups. Data presented as mean ±SEM of two independent experiments with n=2-4 scaffolds per condition. *** indicates a significant difference (p< 0.01; one-way ANOVA (with Tukey post-hoc test) between control and experimental groups).


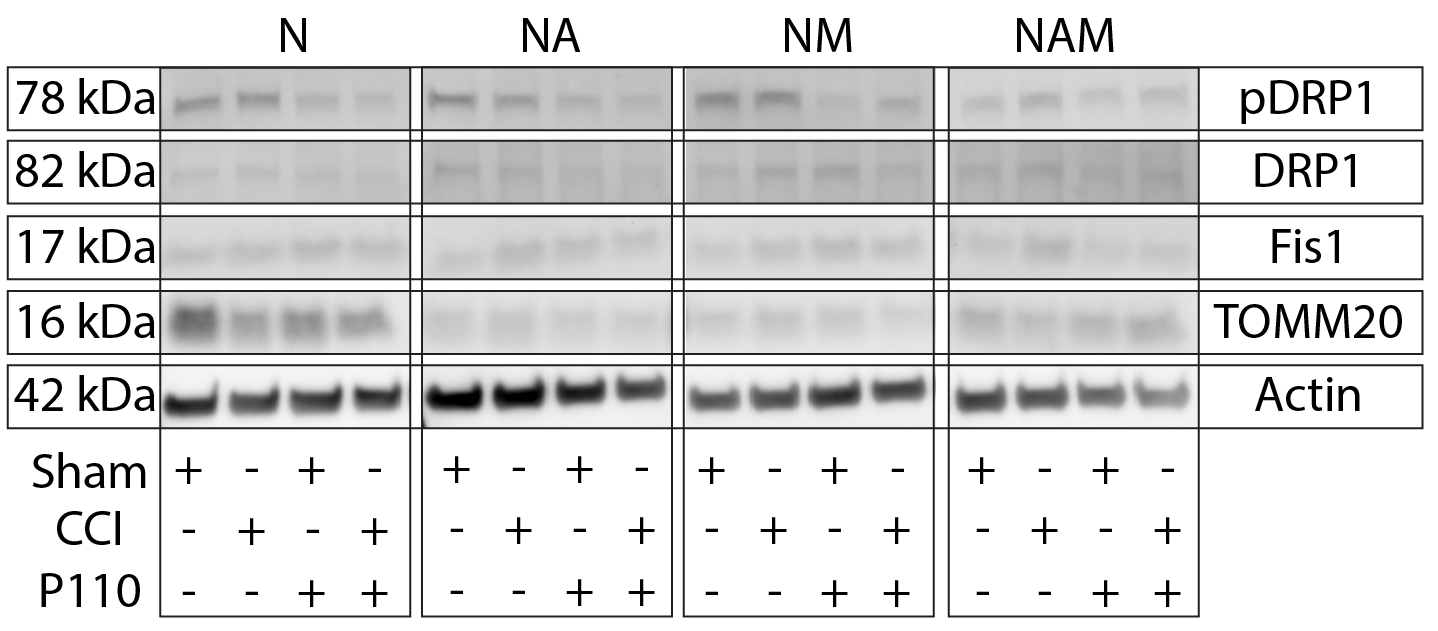


**Supplementary Fig. 13. Injury-induced neurodegeneration in tricultures, but not neuronal monocultures, was associated with Drp1-Fis1 driven mitochondria fission.** In all groups, representative western blots of pDrp1, Drp1, Fis1, and TOMM20 markers. Proteins were isolated from the entire scaffolds without separating the injured area from the penumbra.


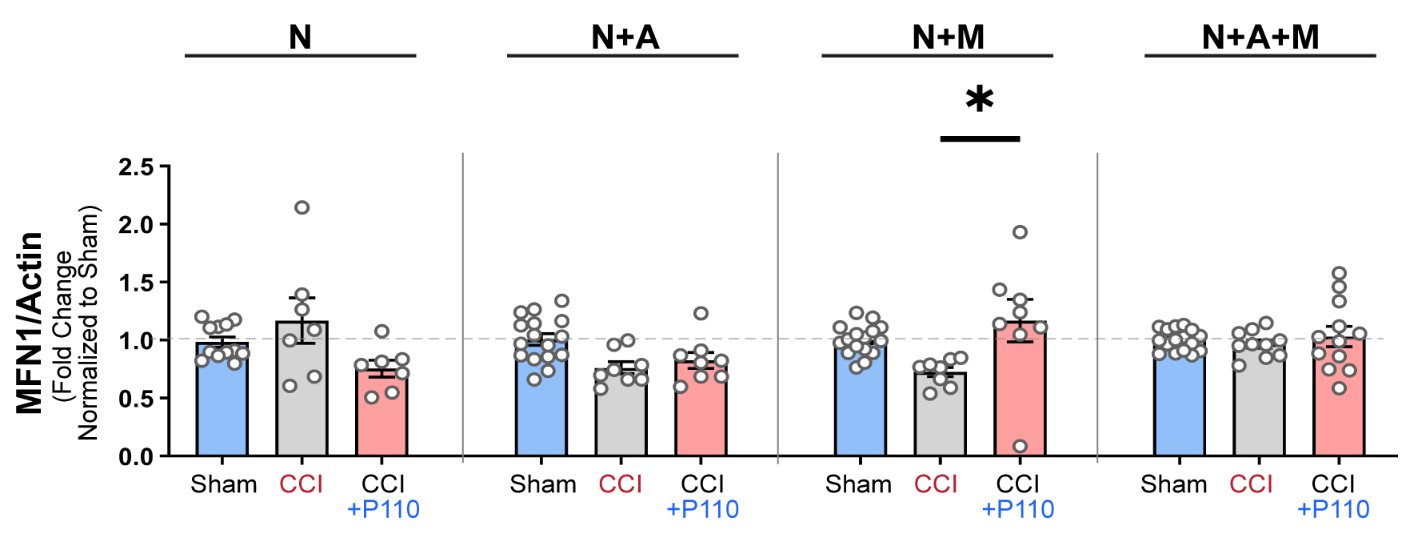


**Supplementary Fig. 14. Secondary neurodegeneration in tricultures was associated with increased mitochondria fission but not fusion.** Mitofusin 1, 2 protein amount quantification of Western Blots 24 hours after injury in control and P110 treated groups. Data presented as mean ±SEM of three independent experiments with n=2-4 scaffolds per condition. *, **, ***, **** indicates significant difference (p<0.05, 0.01, 0.001, 0.0001, respectively; one-way ANOVA (analysis of variance) between control and experimental groups).


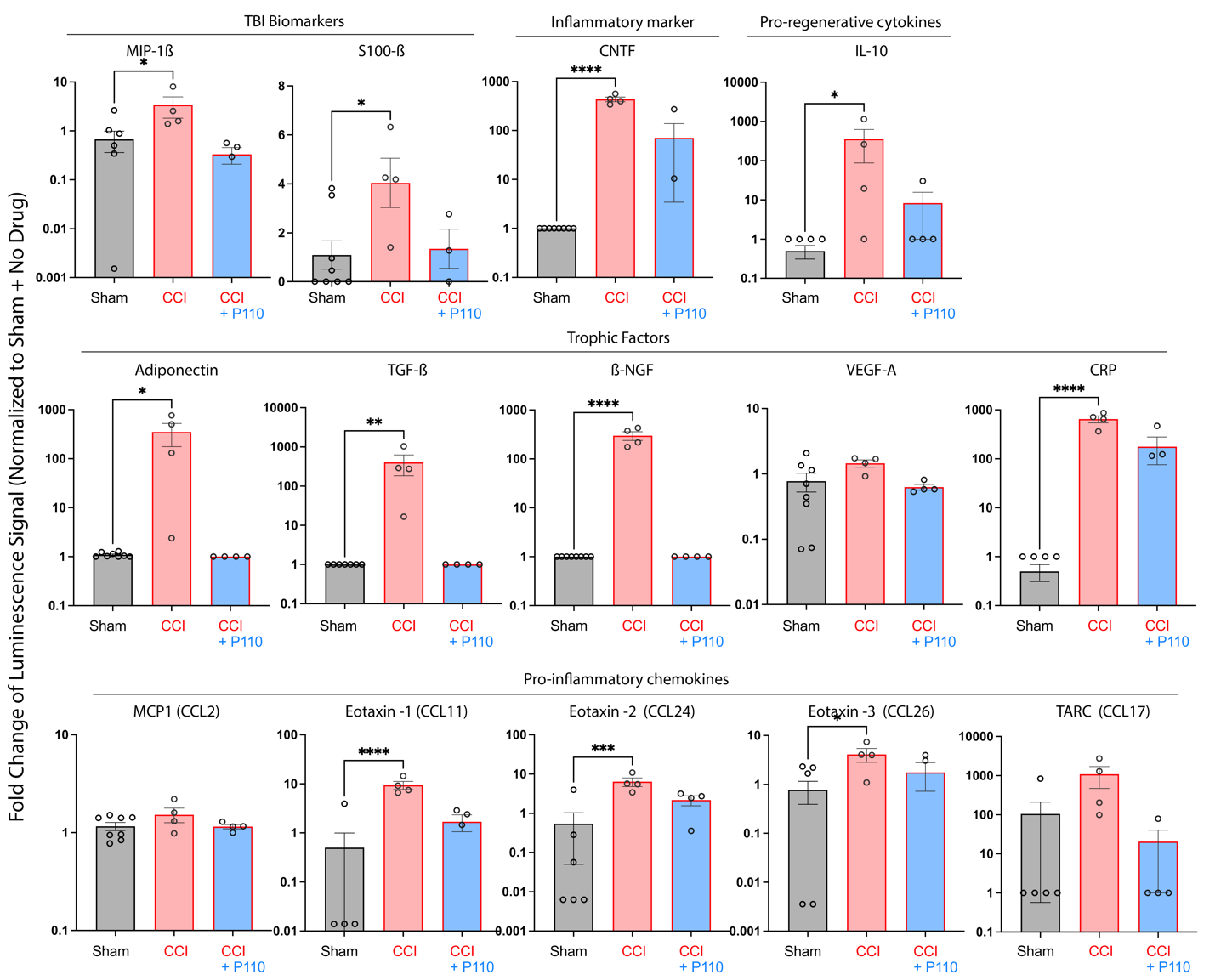


**Supplementary Fig. 15.** **Increased mitochondria fission was associated with neuroinflammation in tricultures 24 hours after the injury.** The production level of TBI biomarkers, pro-inflammatory markers, Pro-regenerative cytokines, Trophic factors, and Pro-Inflammatory chemokines. Data presented as mean ± SEM of four independent experiments with each data point is the average for n=2-4 scaffolds per condition. *, **, ***, **** indicate significant differences (p<0.05, 0.01, 0.001, 0.0001, respectively; two-way ANOVA (analysis of variance) between control and experimental groups).


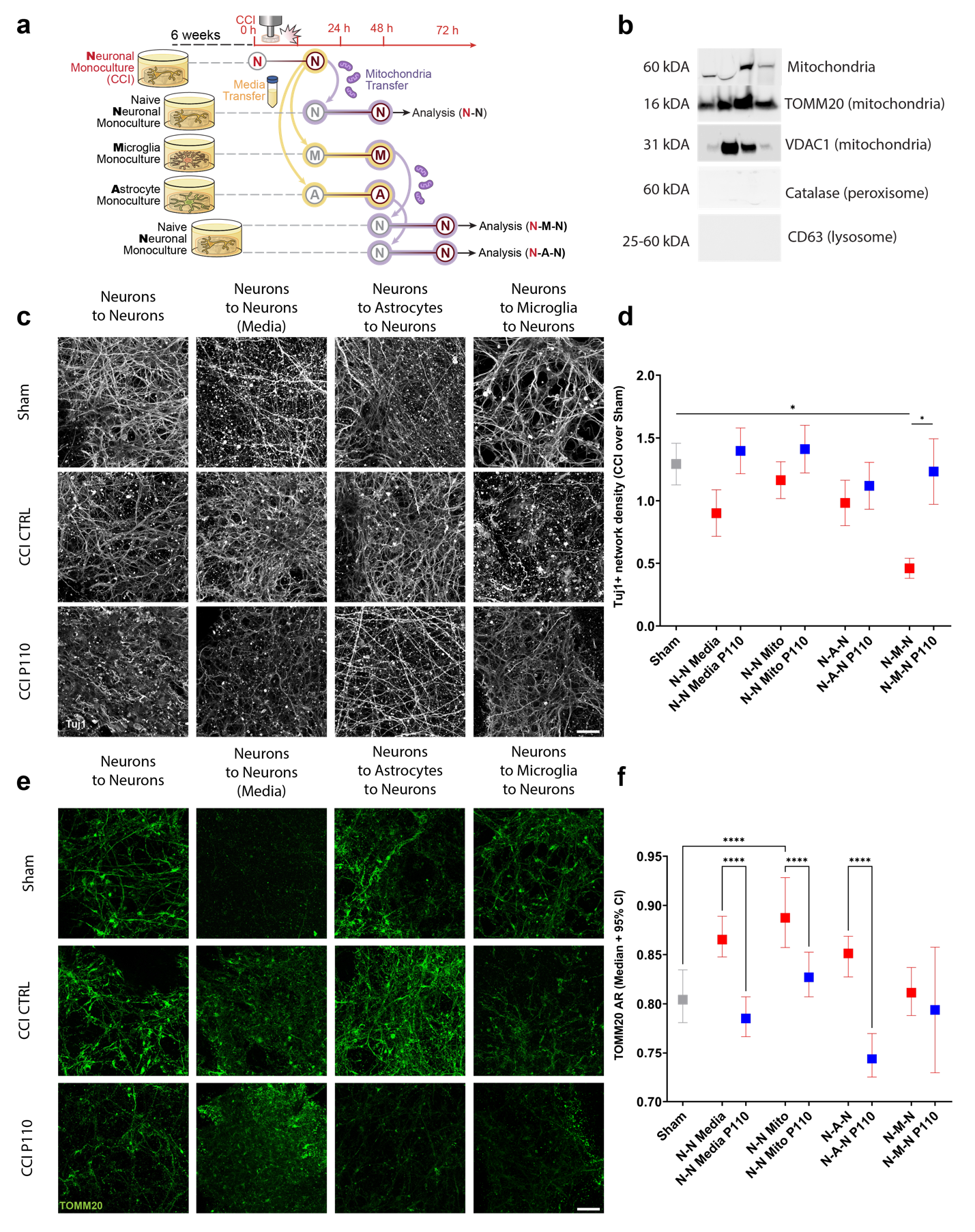


**Supplementary Fig. 16. Mitochondria fragmentation in microglia was crucial for secondary neurodegeneration and neuroinflammation progression post-CCI. a,** Schematic representation of transfer experiments from neurons to neurons, or astrocytes, or microglia. **b,** Western blot analysis of mitochondria-specific proteins expression: anti-mitochondria, TOMM20, and VDAC1 and of peroxisomes (catalase) and lysosomes (CD63) to ensure the purity of mitochondria isolation. Representative images of **c,** Tuj1, and **e,** TOMM20 staining of naïve neurons treated for 24 hours with mitochondria isolated from microglia treated with conditioned media collected from 24h injured neurons with quantification of **d,** Tuj1 positive neuronal network density, and **f,** TOMM20 positive mitochondria aspect ratios. Data presented in **(d, f)** mean ±SEM of n=2-10 scaffolds per condition. *, **, ***, **** indicates significant difference (p<0.05, 0.01, 0.001, 0.0001, respectively; one-way or two-way ANOVA (analysis of variance) between experimental groups). Experiments were replicated at least three times. Scale bar: 50 µm.


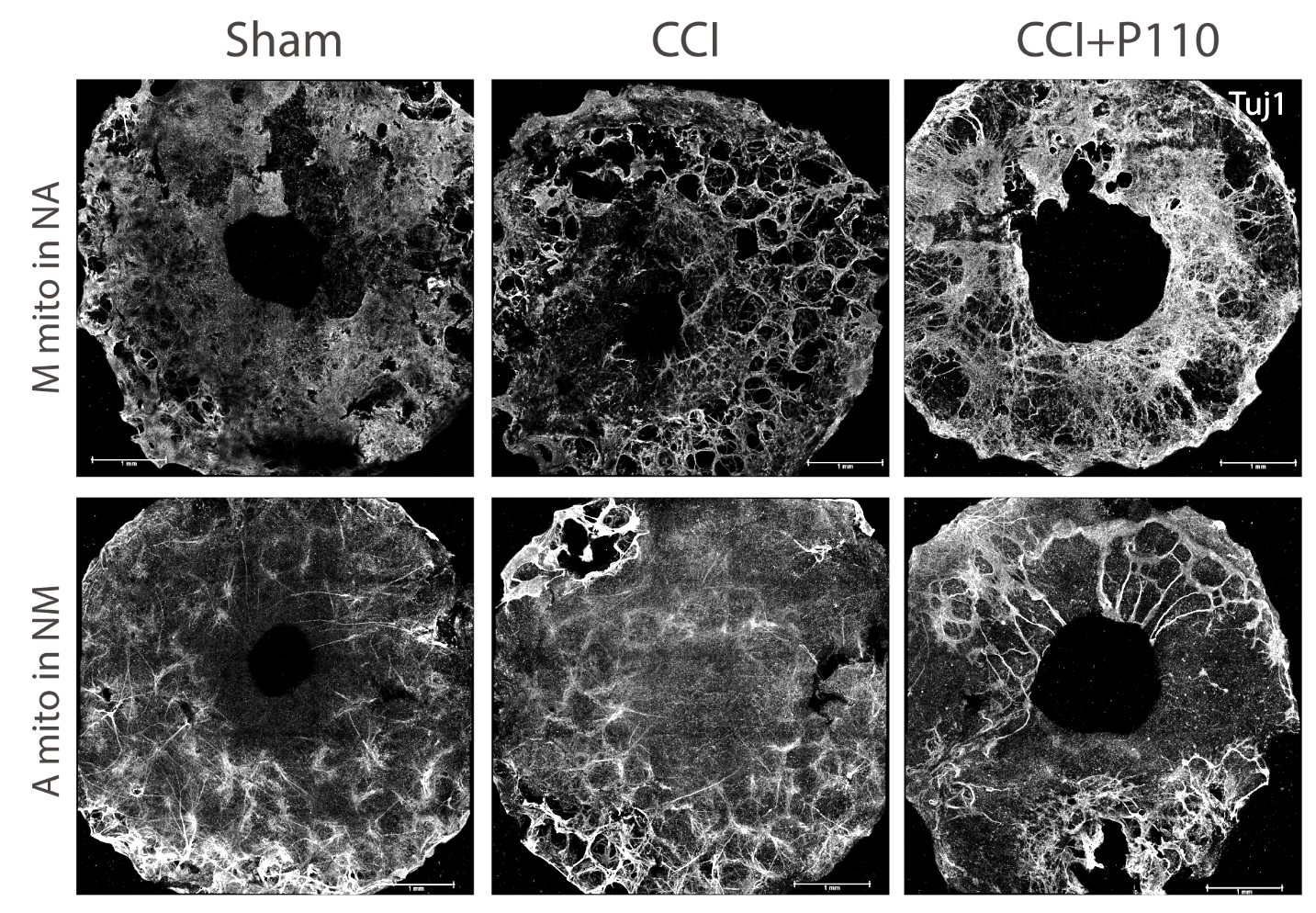


**Supplementary Fig. 17. Mitochondria released from microglia activated by conditioned media from injured neurons induced neuronal network degeneration in naïve co-culture of neurons with astrocytes in mitochondria fission dependent manner, but not by mitochondria released from astrocytes activated by conditioned media from injured neurons induced neuronal network degeneration in naïve co-culture of neurons with microglia.** Representative images of Tuj1 neuronal network staining 24 hours after treatment in NAM groups with and without P110 treatment. Scale bar: 1mm.

**
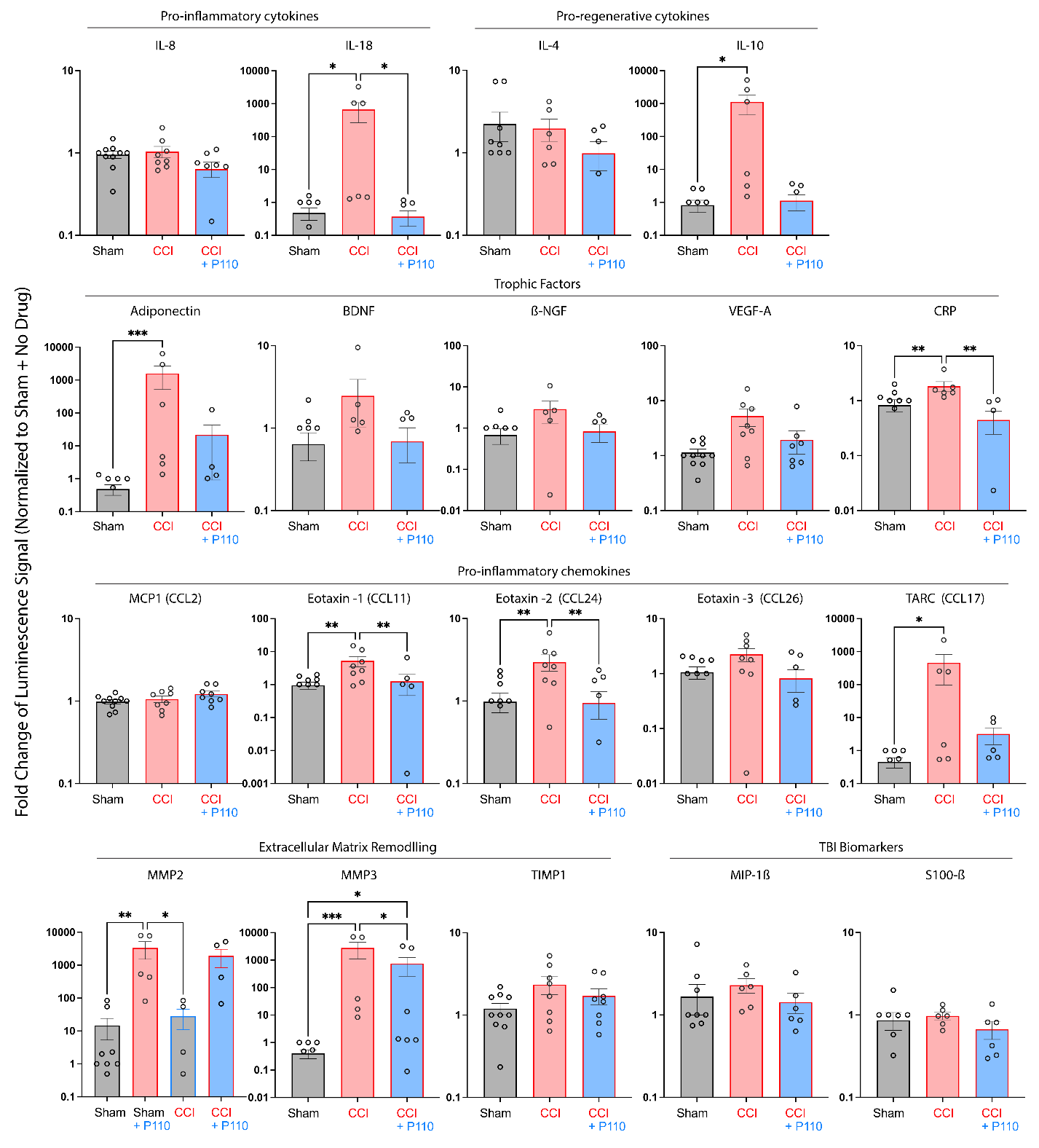
**

**Supplementary Fig. 18.** **Mitochondria dysregulation in microglia was crucial for secondary neurodegeneration and neuroinflammation progression post-CCI.** The production level of Pro-inflammatory and pro-regenerative cytokines, trophic factors, pro-Inflammatory chemokines, extracellular matrix remodeling proteins, and TBI biomarkers. Data presented as mean ± SEM of four independent experiments with each data point is the average for n=2-4 scaffolds per condition. *, **, ***, **** indicate significant differences (p<0.05, 0.01, 0.001, 0.0001, respectively; two-way ANOVA (analysis of variance) between control and experimental groups).


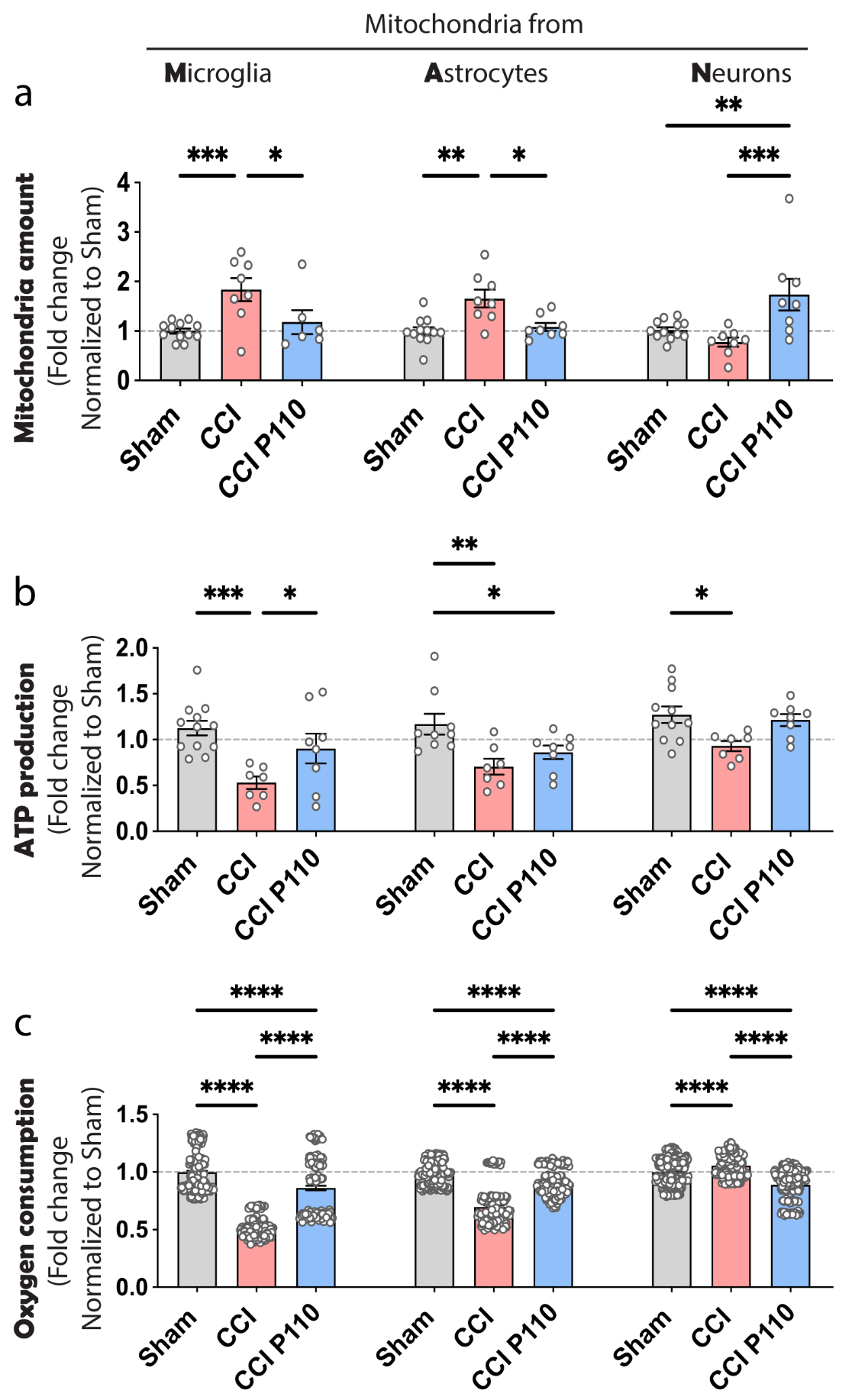


**Supplementary Fig. 19 Conditioned media from injured neurons induced the release of dysfunctional mitochondria from microglia, astrocytes, and neurons. a,** DNA quantification; **b,** ATP production; **c,** Oxygen consumption by the mitochondria released from microglia, astrocytes, and neurons activated by treatment with conditioned media from injured neurons for 24 hours. Data presented as mean ±SEM of n=4-8 scaffolds per condition from 2 independent experiments. *, **, ***, **** indicates significant difference (p<0.05, 0.01, 0.001, 0.0001 respectively; one-way or two-way ANOVA (with Tukey’s post-hoc test) between experimental groups).


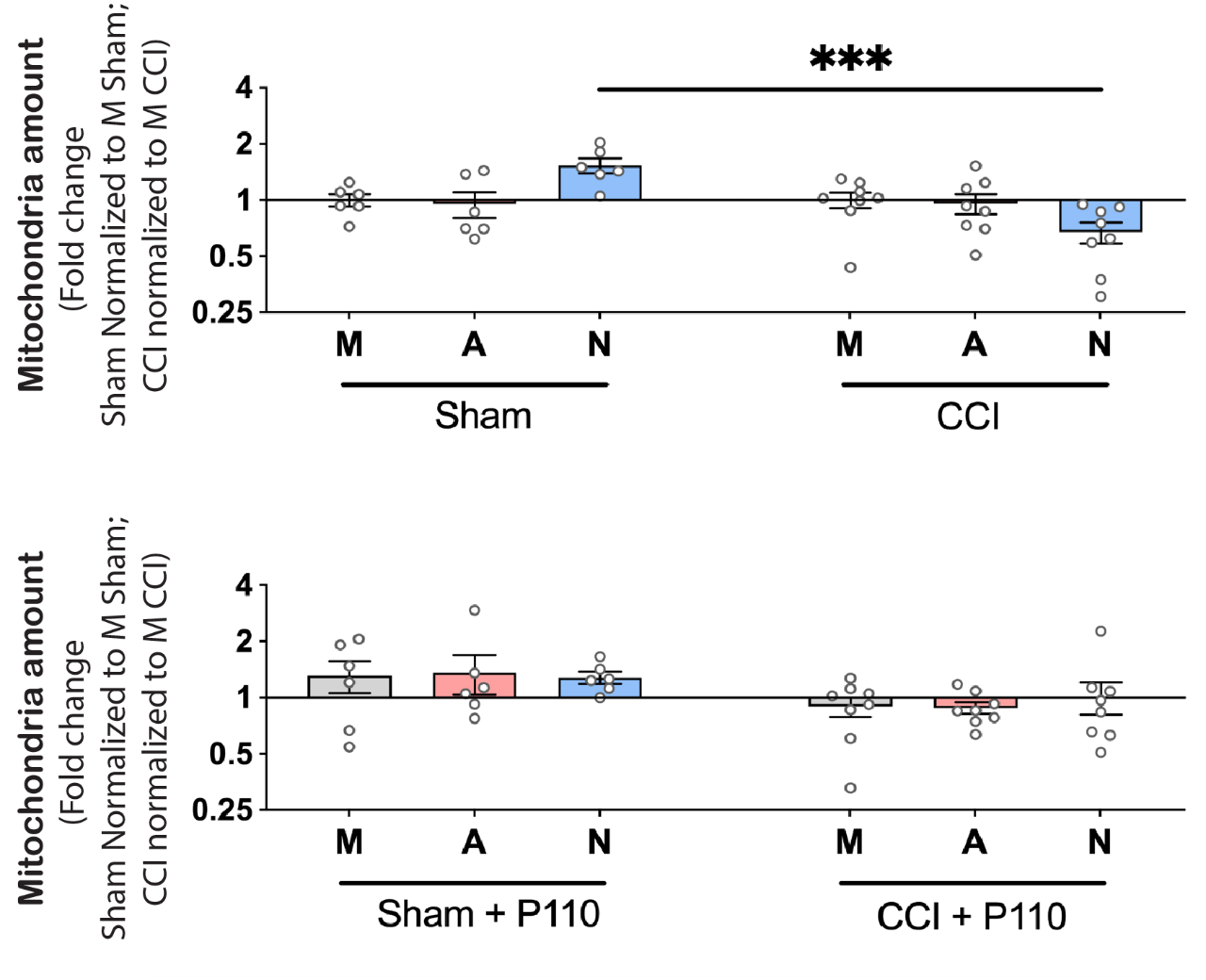


**Supplementary Fig. 20.** **Activated** **naïve neurons re-uptake dysfunctional mitochondria from the extracellular environment.** DNA quantification of mitochondria released from activated by treatment with conditioned media neurons for 24 hours. Data presented as mean ±SEM of n=6-8 scaffolds per condition from 2 independent experiments. *** indicates a significant difference (p< 0.001; one-way or two-way ANOVA (with Tukey’s post-hoc test) between experimental groups).


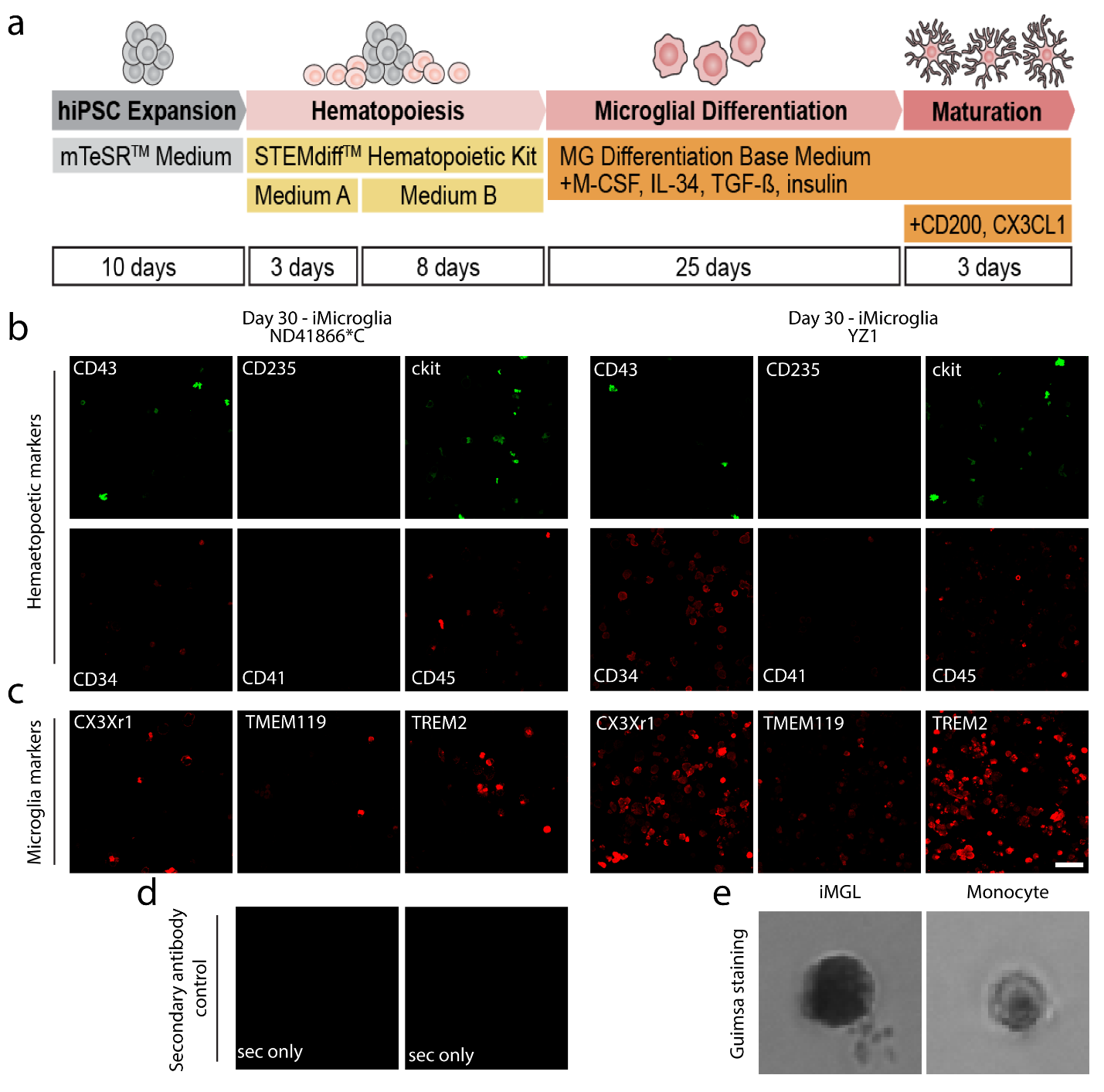


**Supplementary Fig. 21. iPSC-derived microglia characterization.** **a,** Schematic representation of the microglia differentiation protocol from induced pluripotent stem cells of two healthy donors (YZ1 and ND41866*C). **b,** Hematopoietic markers: CD43, CD34, CD235, CD41, and c-kit; **c,** Microglial markers: CD45, CX3CR1, TMEM119, TREM2; **d,** Secondary antibody control; **e,** Guimsa staining in comparison to human monocytes. Scale bar: 50μm.


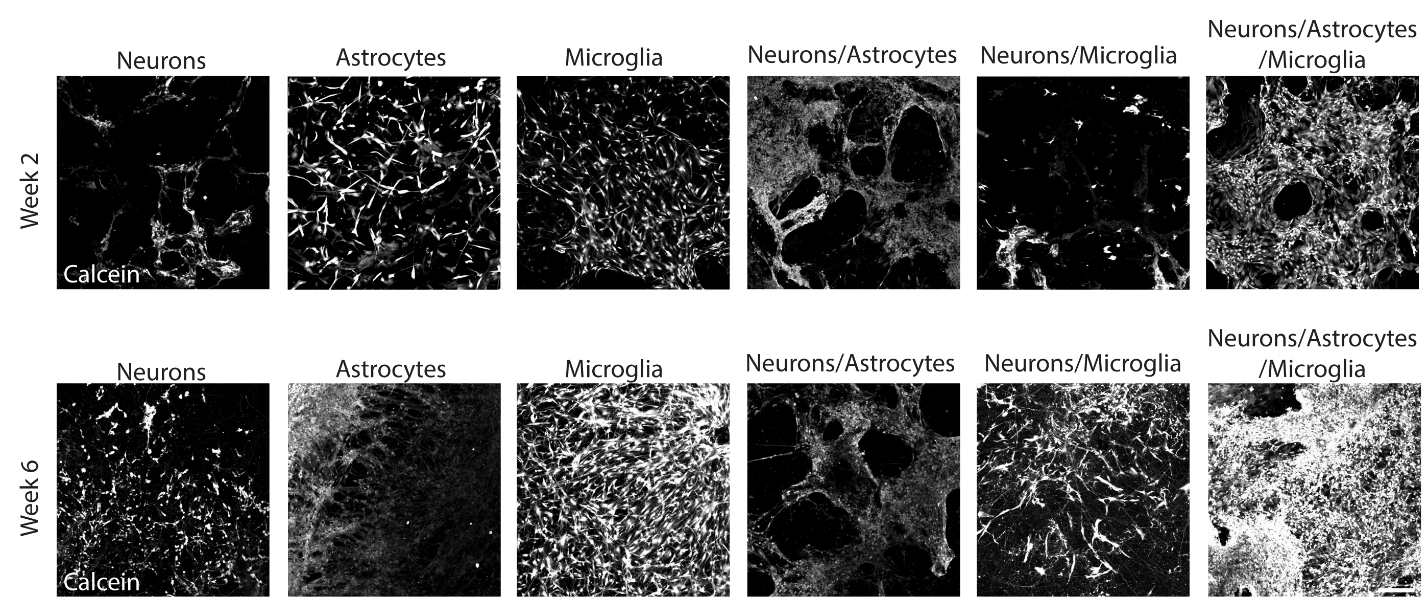


**Supplementary Fig. 22. Tricultures with iPSC-derived microglia were alive at 6 weeks before controlled cortical impact injuries.** Calcein staining of live samples at 2 and 6 weeks after seeding. Scale bar: 100μm.


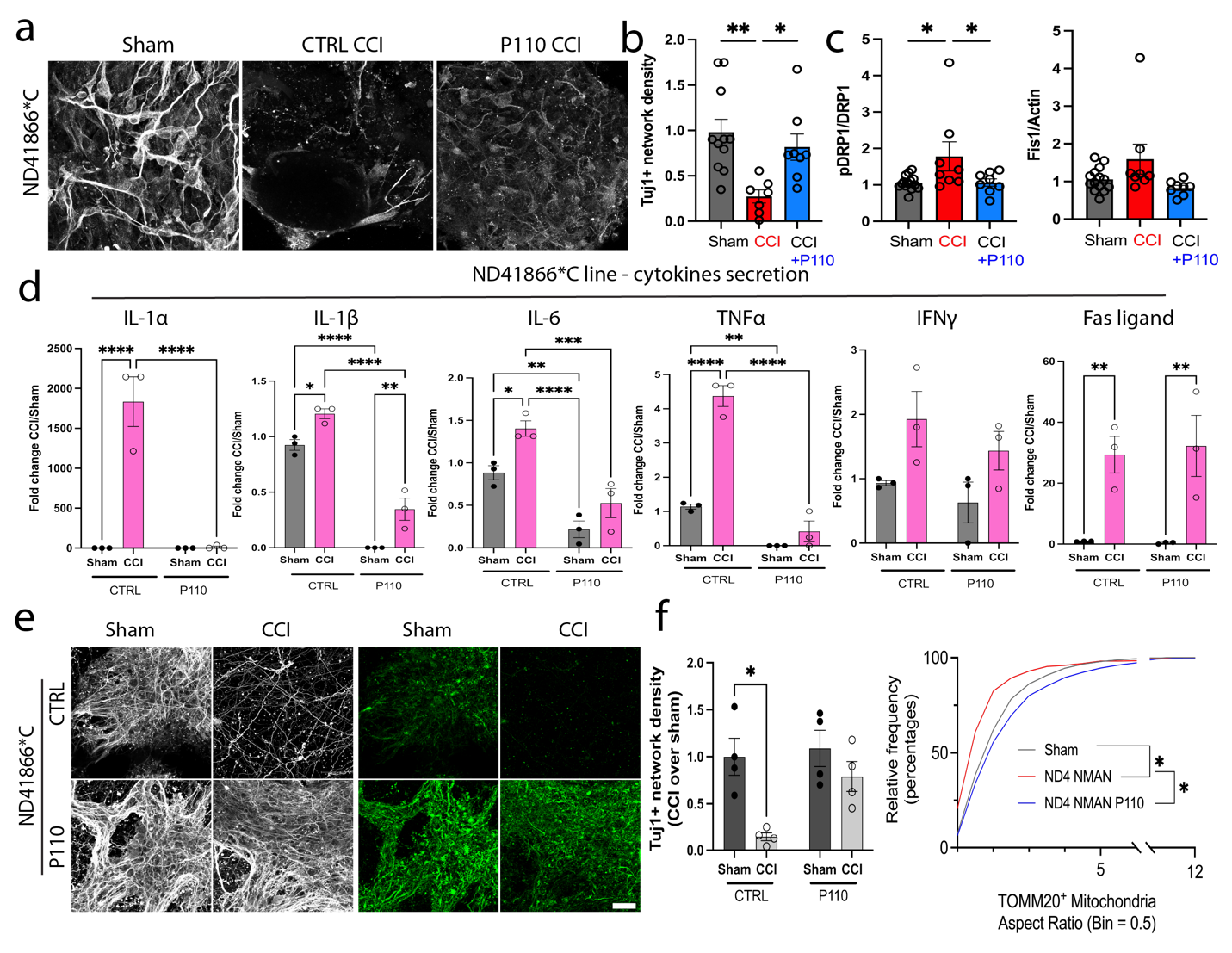


**Supplementary Fig. 23. Fragmented mitochondria released from iPSCs-derived microglia (ND41866*C line from a healthy donor) induced neurodegeneration and neuroinflammation progression 24 hours after controlled cortical impact injury. a,** Representative images of Tuj1 network density in tricultures with iPSC-derived microglia. **b,** Quantification of the Tuj1 neuronal network density in control and P110 treated groups of NAM group. **c,** Mitochondria fission associated pDRP1 and FIS1 proteins expression in control and P110 treated NAM groups. **d,** Inflammatory cytokines secretion post-CCI in YZ1 triculture groups. **e,** Representative images, and **f,** Quantifications of Tuj1 neuronal and TOMM20 mitochondria networks in naïve neurons treated with mitochondria isolated from astrocytes activated with mitochondria isolated from YZ1 microglia treated with conditioned media collected from 24h injured neurons. Data presented as mean ±SEM of n=2-10 scaffolds per condition. *, **, ***, **** indicates significant differences (p<0.05, 0.01, 0.001, 0.0001 respectively; two-way ANOVA (analysis of variance) or one-way ANOVA between control and experimental groups). Experiments were replicated at least three times. Scale bar: 50μm


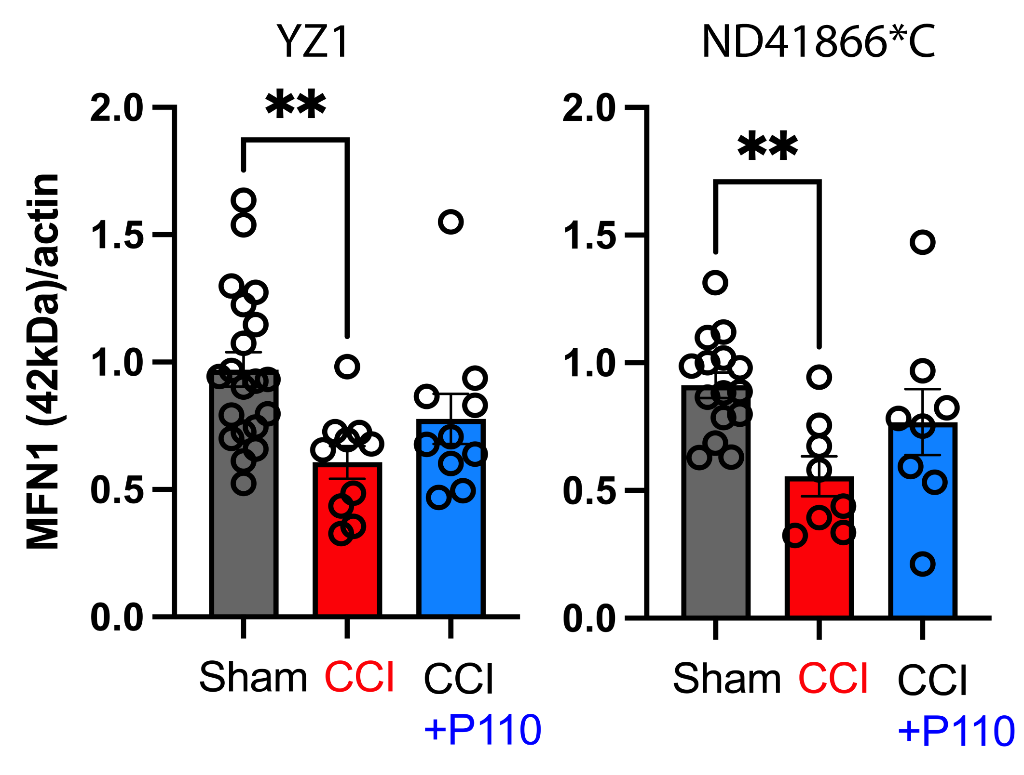


**Supplementary Fig. 24.** **Secondary neurodegeneration in tricultures with iPSC-derived microglia (both donors) was associated with decreased mitochondria fusion.** Mitofusin 1, 2 (42 kDa isoform) protein amount quantification of Western blots 24 hours after injury. Data presented as mean ±SEM of three independent experiments with n=2-4 scaffolds per condition. *, **, ***, **** indicates significant difference (p<0.05, 0.01, 0.001, 0.0001, respectively; two-way ANOVA (analysis of variance) between control and experimental groups).


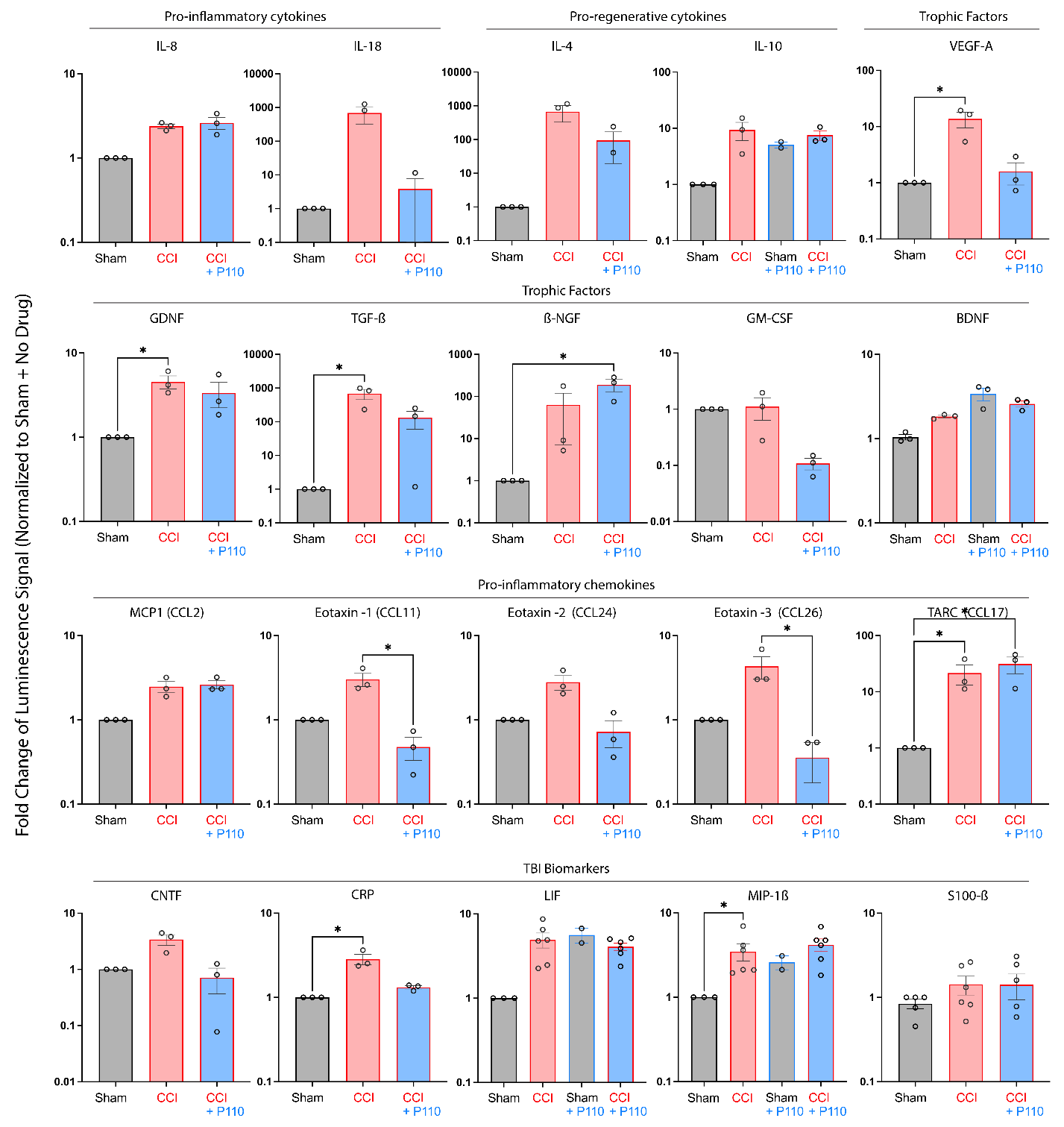


**Supplementary Fig. 25.** **Increased mitochondria fission was associated with neuroinflammation in tricultures with iPSC-derived microglia (YZ1 line) 24 hours after the injury.** The production level of: Pro-inflammatory and pro-regenerative cytokines, trophic factors, pro-Inflammatory chemokines, and TBI biomarkers. Data presented as mean ± SEM of four independent experiments with each data point is the average for n=2-4 scaffolds per condition. *, **, ***, **** indicate significant differences (p<0.05, 0.01, 0.001, 0.0001, respectively; two-way ANOVA (analysis of variance) between control and experimental groups).


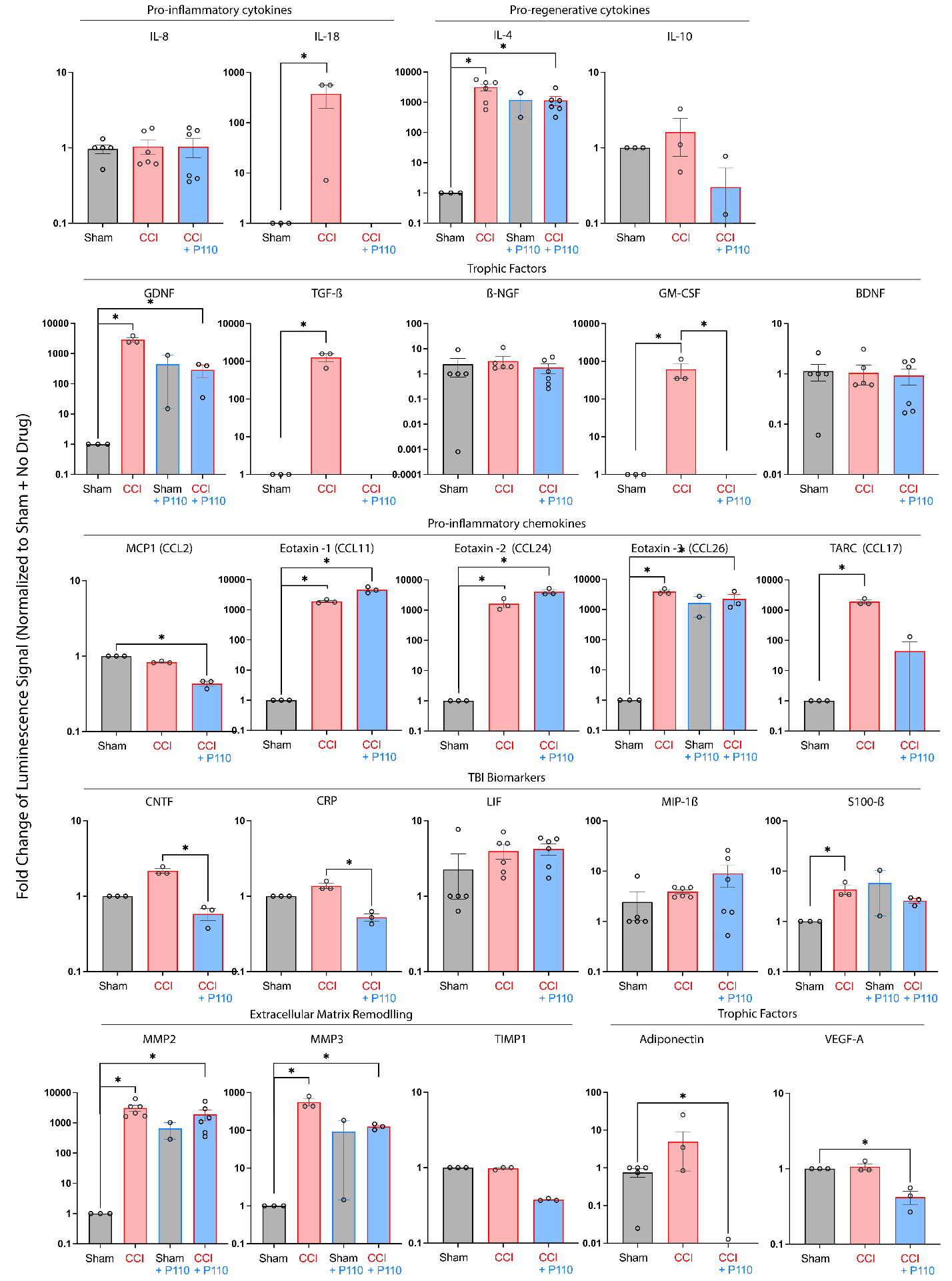


**Supplementary Fig. 26.** **Increased mitochondria fission was associated with neuroinflammation in tricultures with iPSC-derived microglia (ND41866*C line) 24 hours after the injury.** The production level of: Pro-inflammatory and pro-regenerative cytokines, trophic factors, pro-Inflammatory chemokines, and TBI biomarkers. Data presented as mean ± SEM of four independent experiments with each data point is the average for n=2-4 scaffolds per condition. *, **, ***, **** indicate significant differences (p<0.05, 0.01, 0.001, 0.0001, respectively; two-way ANOVA (analysis of variance) between control and experimental groups).


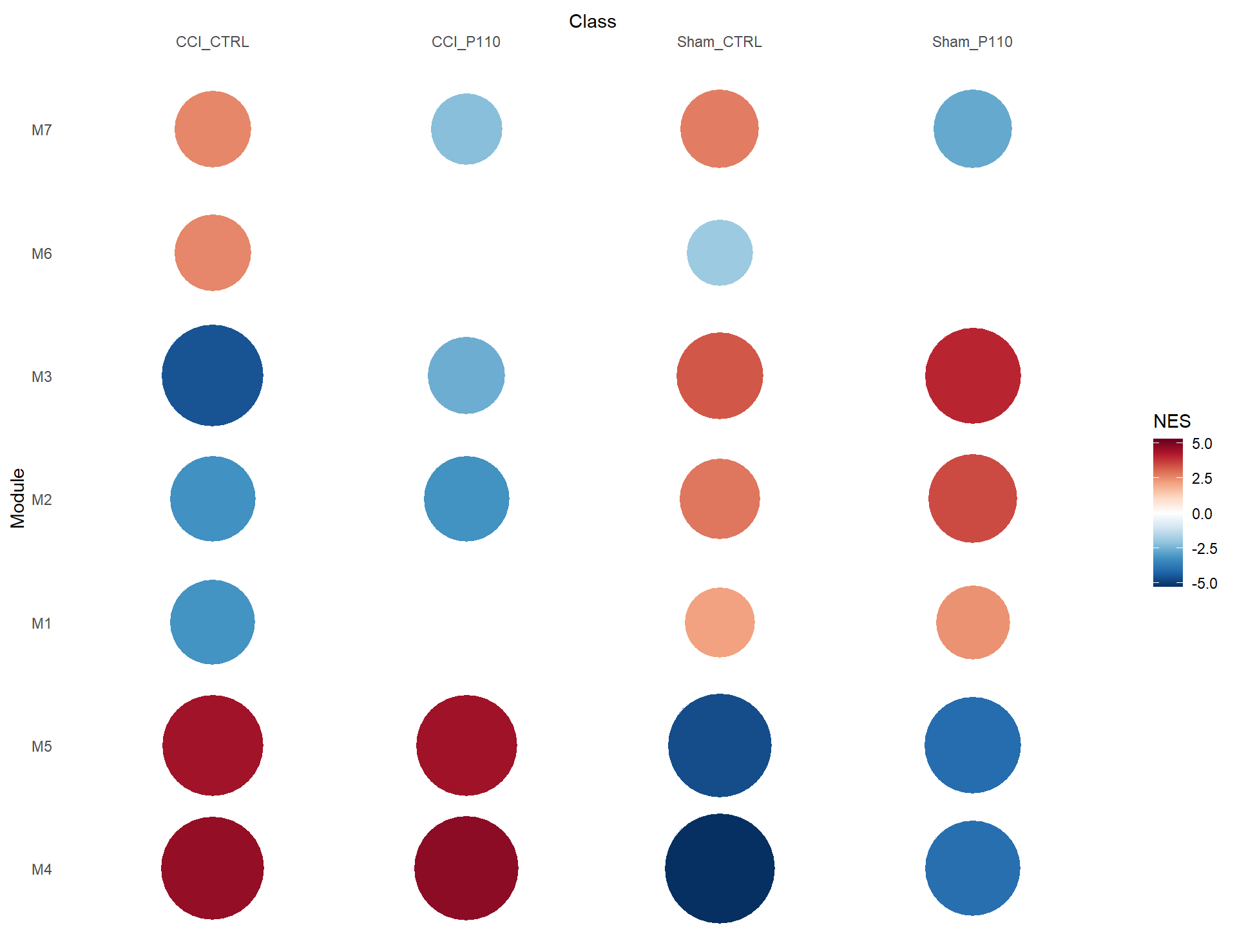


**Supplementary Fig. 27.** **Co-expression network analysis revealed a significant shift in gene expression associated with cellular proliferation (Module 5), extracellular matrix remodeling (Modules 2, 4), interferon signaling (Module 6), and neuronal systems (Modules 1, 3) in human 3D triculture model in response to contusion injury.** P110 treatment removed the positive association of contusion injury with interferon-associated inflammation in Module 6, while In Module 7, P110 treatment was negatively associated with collagen formation and assembly but not injury. In modules 1-3, gene expression was negatively associated with a contusion, while in Modules 4-5, the association was positive. Changes in differentially expressed genes 24 hours post-contusion injury in human 3D triculture model composed of induced neural stem cells derived neurons, primary astrocytes, and iPSC-derived microglia from two healthy donors YZ1 and ND418664*F (n=6).


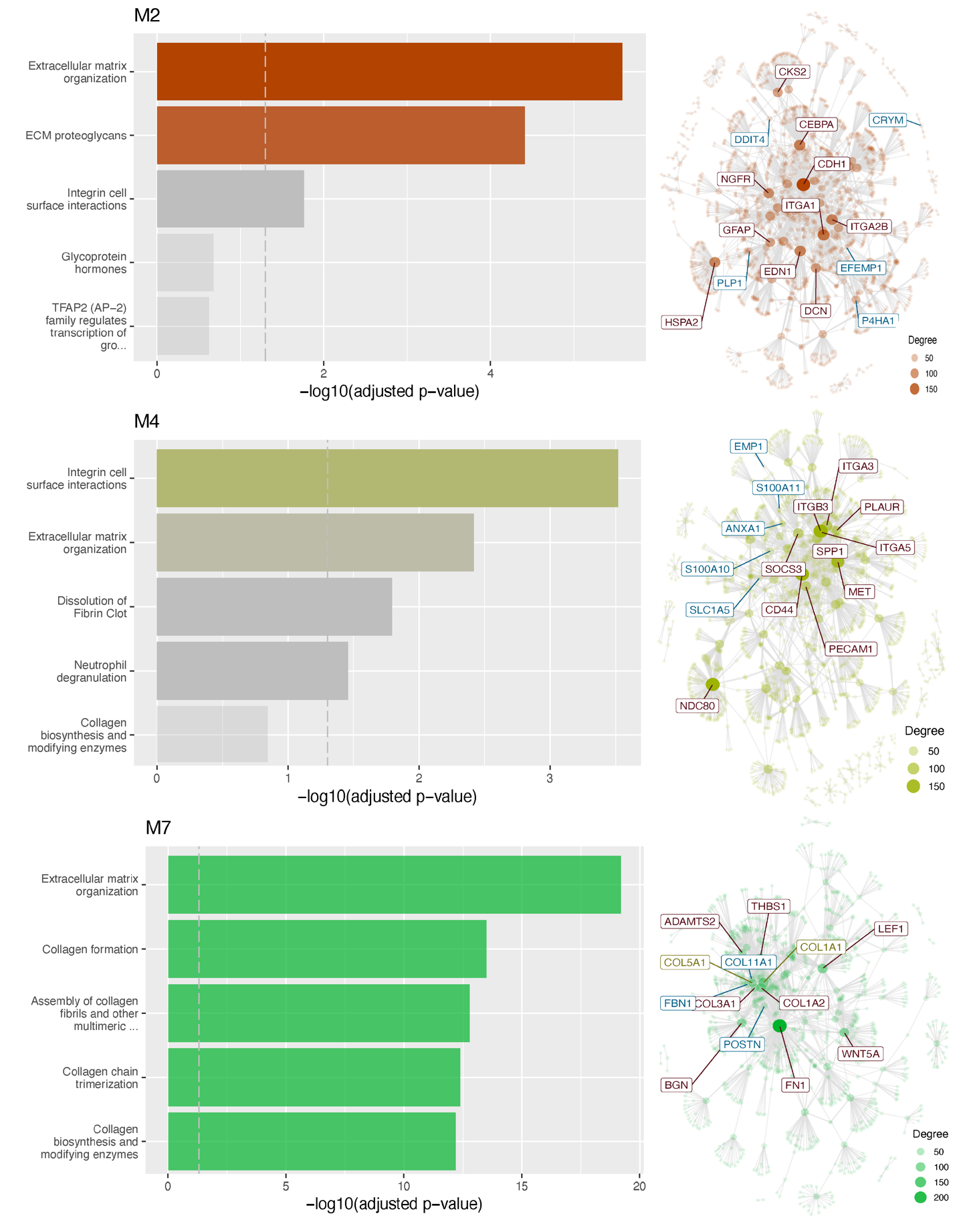
**Supplementary Fig. 28. Contusion injury-induced shift in the gene expression of extracellular matrix components in Modules 2 and 4.** Gene set enrichment analysis revealed distinct expression modules associated with M2 – ECM proteoglycans (negatively associated with a contusion in CTRL and P110); M4 – integrin cell surface interactions (positively associated with a contusion in CTRL and P110); M7 – collagen formation and assembly (positively associated with P110 treatment, but not the injury) and their corresponding regulators. Changes in differentially expressed genes 24 hours post-contusion injury in human 3D triculture model composed of induced neural stem cells derived neurons, primary astrocytes, and iPSC-derived microglia from two healthy donors YZ1 and ND418664*F (n=6).


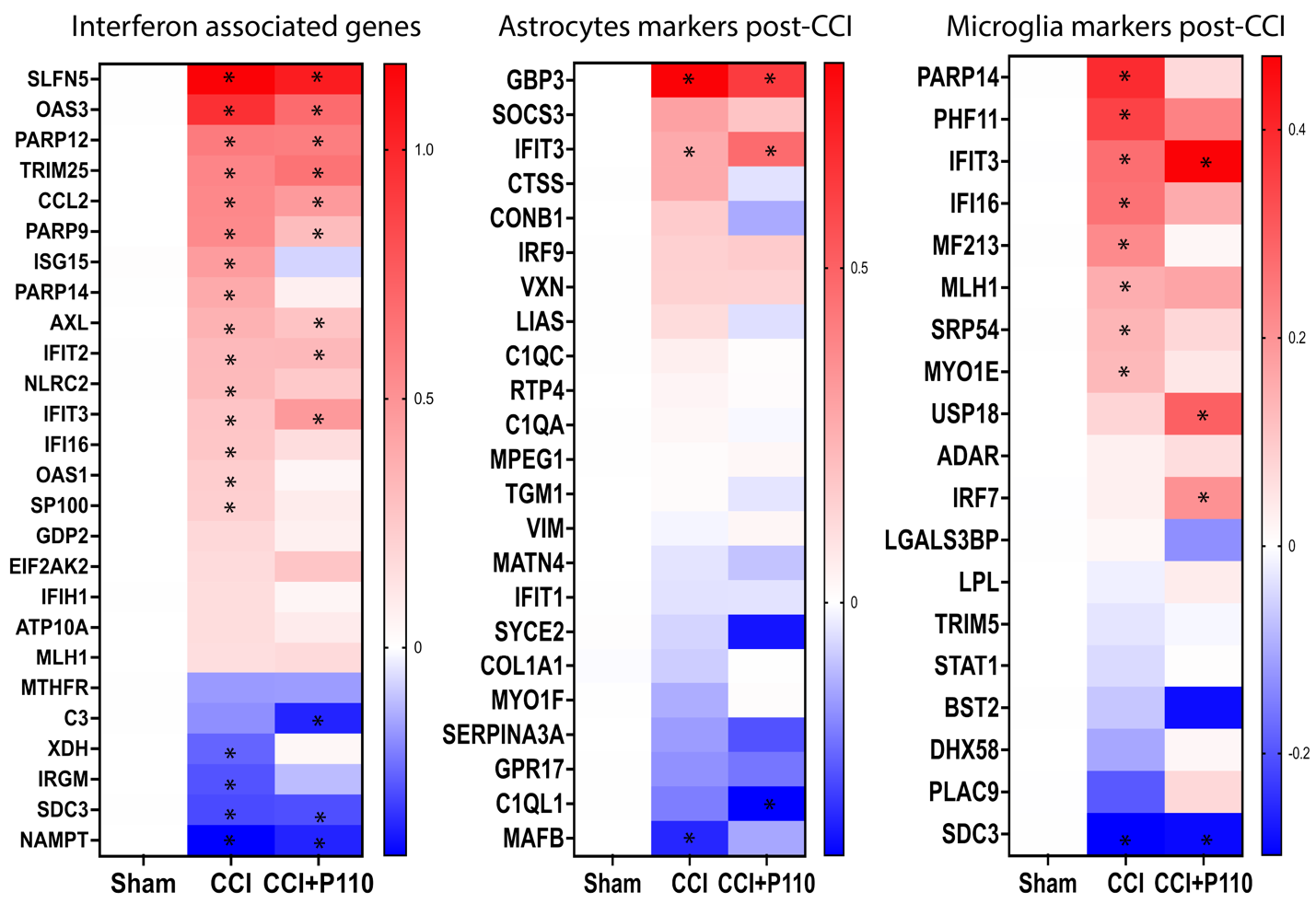


**Supplementary Fig. 29**. **Contusion injury induces activation of microglia-driven interferon-associated inflammation.** Differentially expressed genes in sham (sham vs sham P110), CCI (CCI vs sham), and CCI+P110 (CCI+P110 vs Sham+110) groups were detected (abs(Log2(Fold change))> 0.585 and q < 0.01). Changes in differentially expressed genes 24 hours post-contusion injury in human 3D triculture model composed of induced neural stem cells derived neurons, primary astrocytes, and iPSC-derived microglia from two healthy donors YZ1 and ND418664*F (n=6).


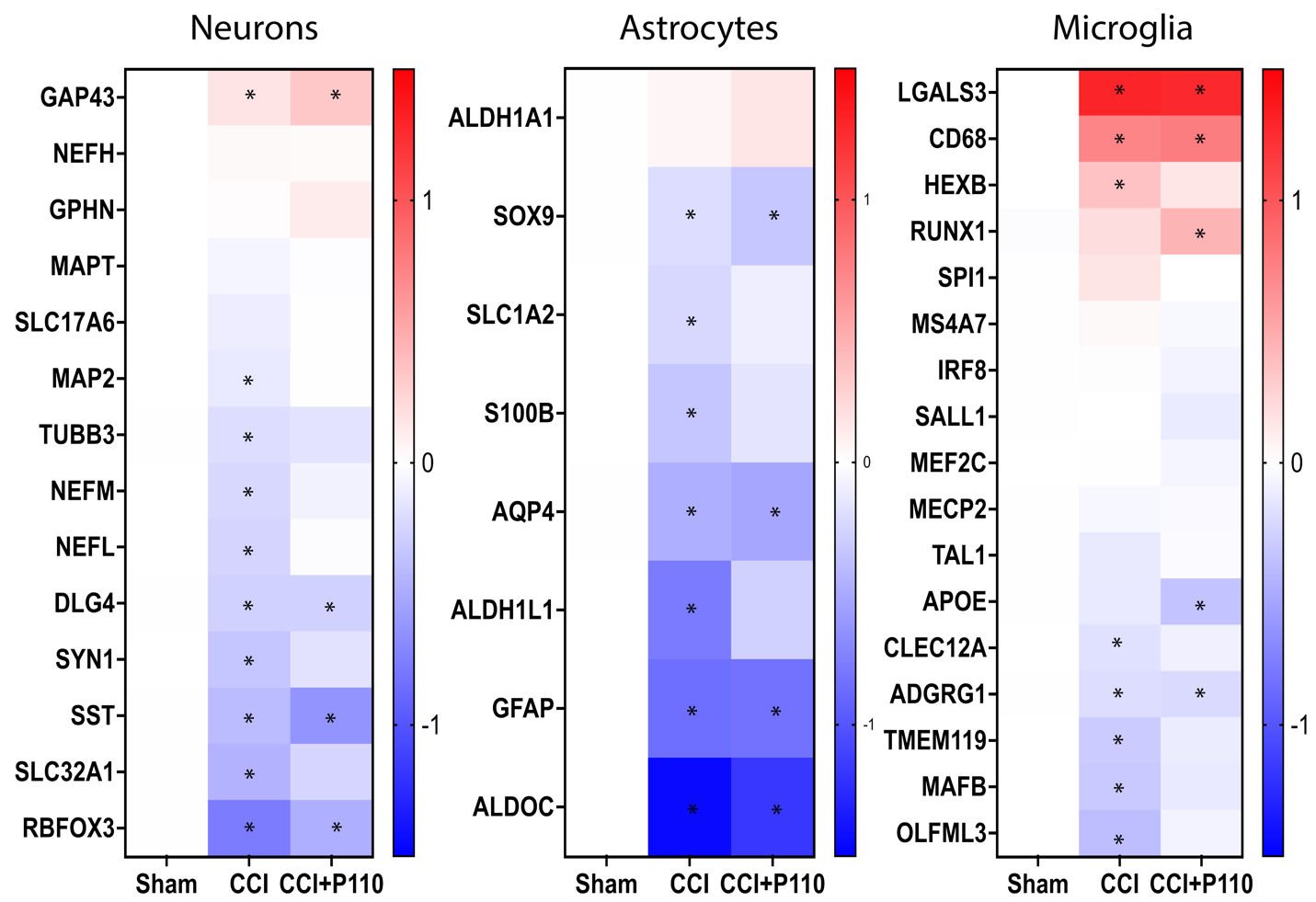


**Supplementary Fig. 30**. **Contusion injury induces a negative shift in neuronal and astrocytic pan-markers but a positive shift in microglial.** Differentially expressed genes in sham (sham vs sham P110), CCI (CCI vs sham), and CCI+P110 (CCI+P110 vs Sham+110) groups were detected (abs(Log2(Fold change))> 0.585 and q < 0.01). Changes in differentially expressed genes 24 hours post-contusion injury in human 3D triculture model composed of induced neural stem cells derived neurons, primary astrocytes, and iPSC-derived microglia from two healthy donors YZ1 and ND418664*F (n=6).


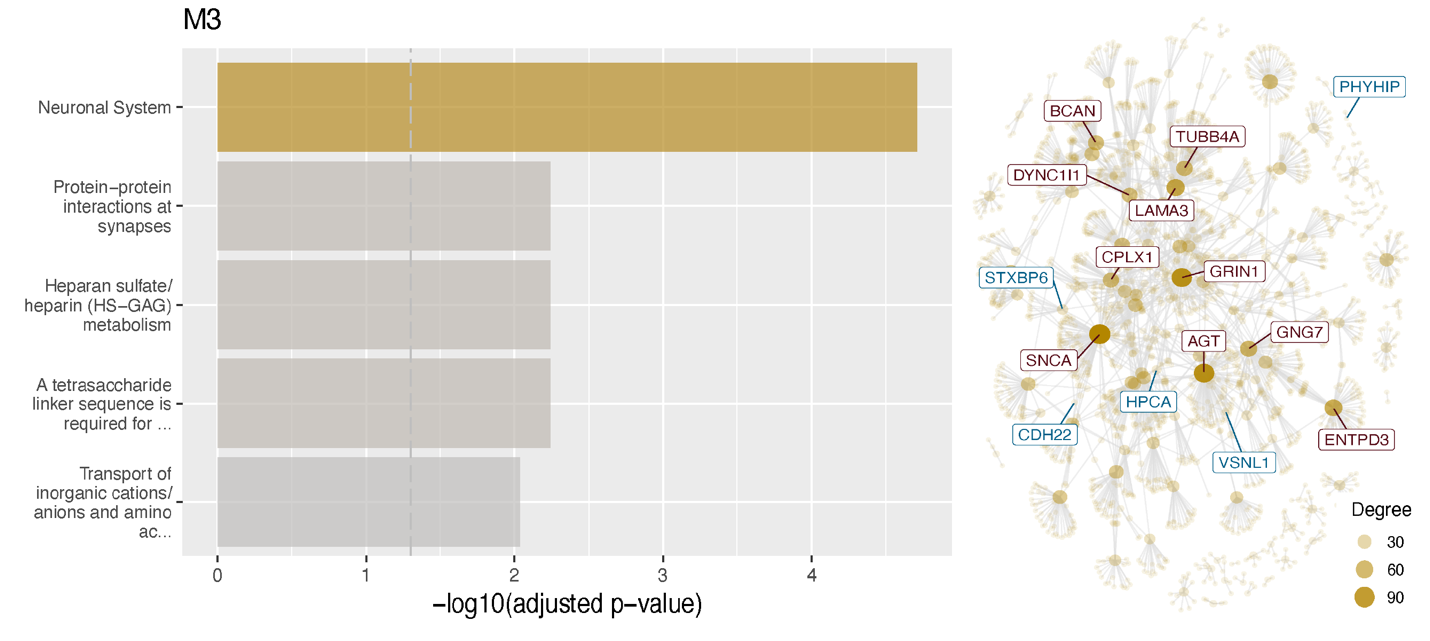
**Supplementary Fig. 31. Contusion injury-induced shift in the gene expression of extracellular matrix components.** Gene set enrichment analysis revealed distinct expression modules associated with M3 – neuronal systems (negatively associated with a contusion in both CTRL and P110 groups) and their gene - regulators. Changes in differentially expressed genes 24 hours post-contusion injury in human 3D triculture model composed of induced neural stem cells derived neurons, primary astrocytes, and iPSC-derived microglia from two healthy donors YZ1 and ND418664*F (n=6).


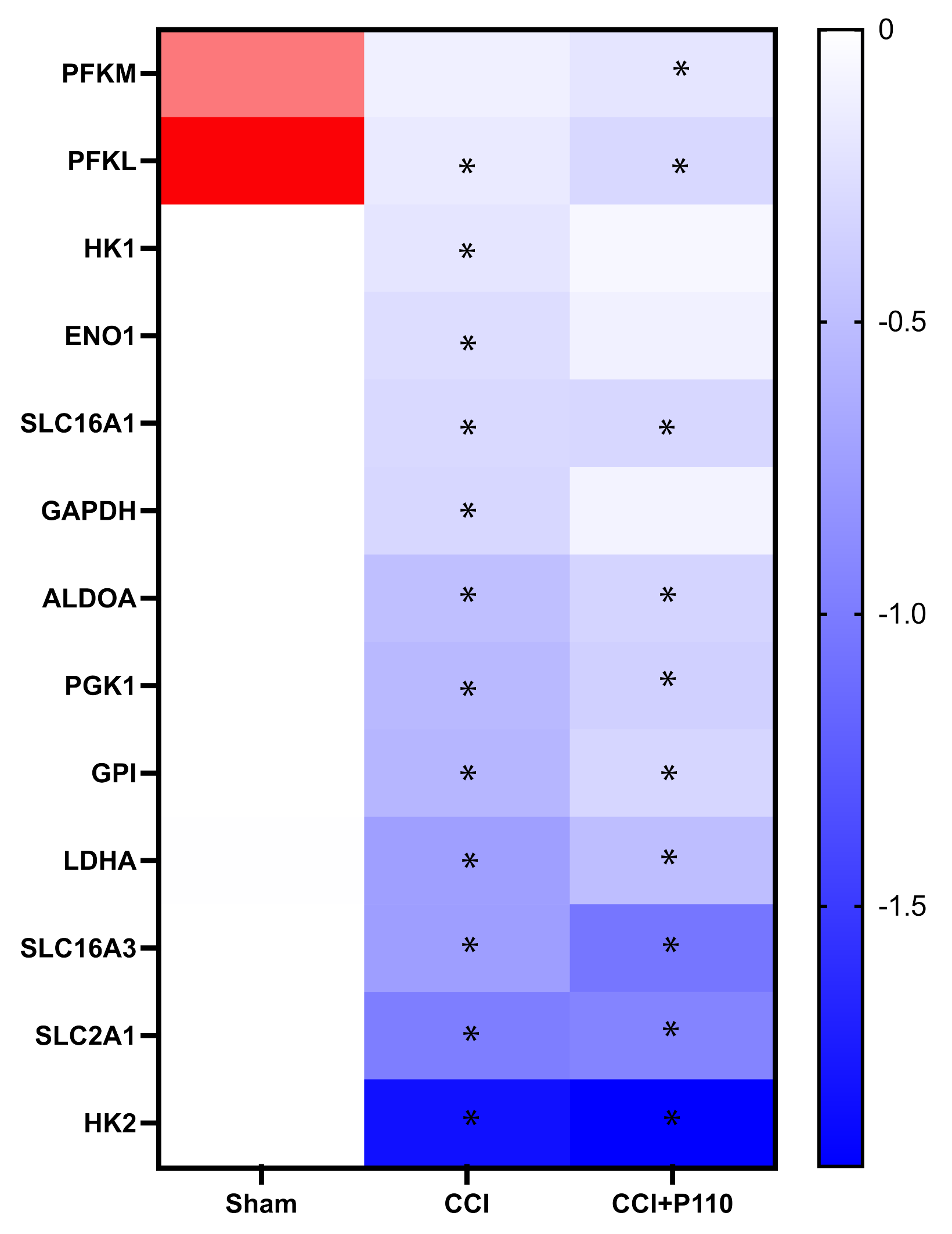


**Supplementary Fig. 32. Contusion injury induces glycolysis downregulation in the short term after the injury.** Differentially expressed genes in sham (sham vs sham P110), CCI (CCI vs sham), and CCI+P110 (CCI+P110 vs Sham+110) groups were detected (abs(Log2(Fold change))> 0.585 and q < 0.01). Changes in differentially expressed genes 24 hours post-contusion injury in human 3D triculture model composed of induced neural stem cells derived neurons, primary astrocytes, and iPSC-derived microglia from two healthy donors YZ1 and ND418664*F (n=6).


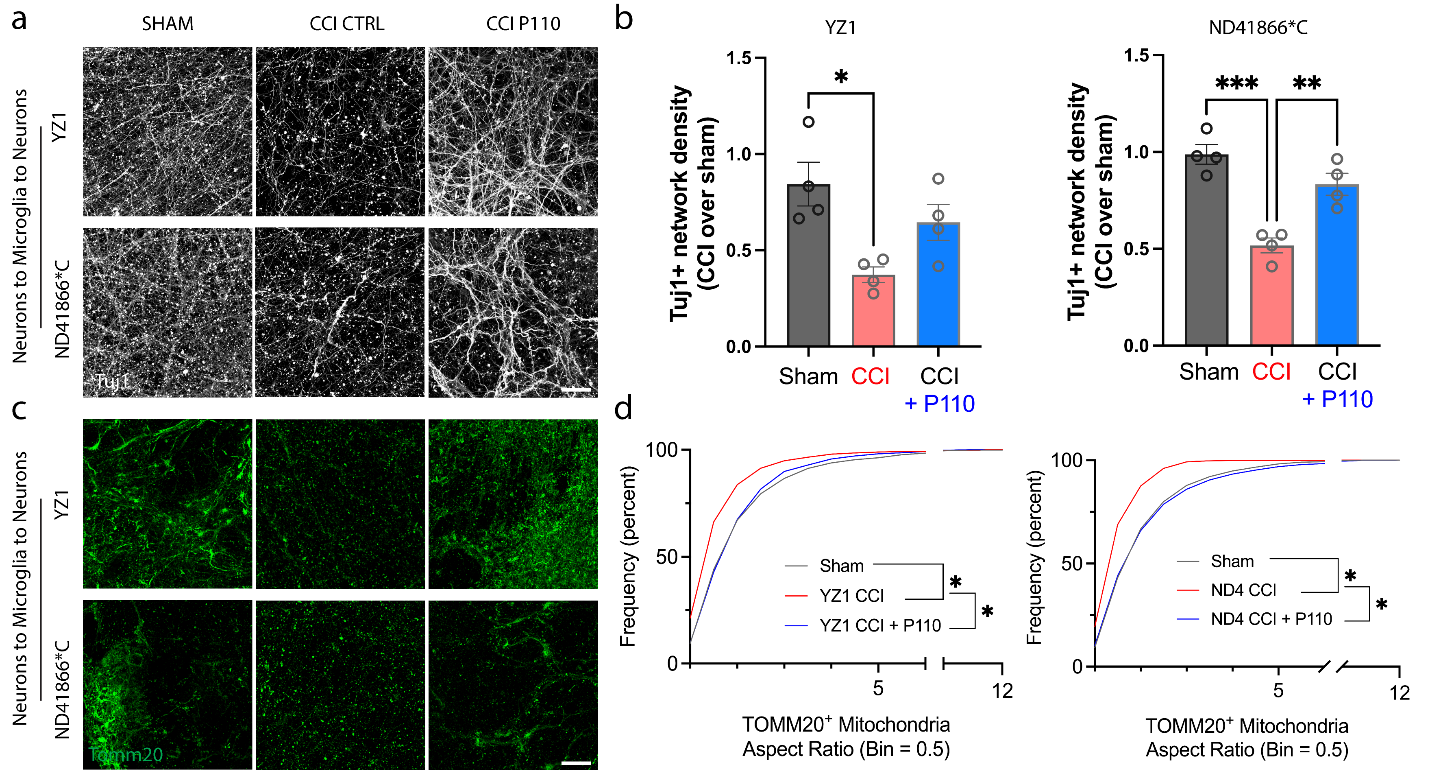


**Supplementary Fig. 33. Fragmented mitochondria released from iPSCs-derived microglia (ND41866*C line from a healthy donor) induced neurodegeneration and neuroinflammation progression 24 hours after controlled cortical impact injury.** Representative images of **a,** Tuj1 and **c,** TOMM20 staining of naïve neurons treated for 24 hours with mitochondria isolated from microglia treated with conditioned media collected from 24h injured neurons with quantification of **b,** Tuj1 positive neuronal network density and **d,** TOMM20 positive mitochondria aspect ratios. Data presented in **(b, d)** mean ±SEM of n=2-10 scaffolds per condition. *, **, ***, **** indicates significant difference (p<0.05, 0.01, 0.001, 0.0001, respectively; one-way or two-way ANOVA (analysis of variance) between experimental groups). Experiments were replicated at least three times. Scale bar: 50 µm.

**
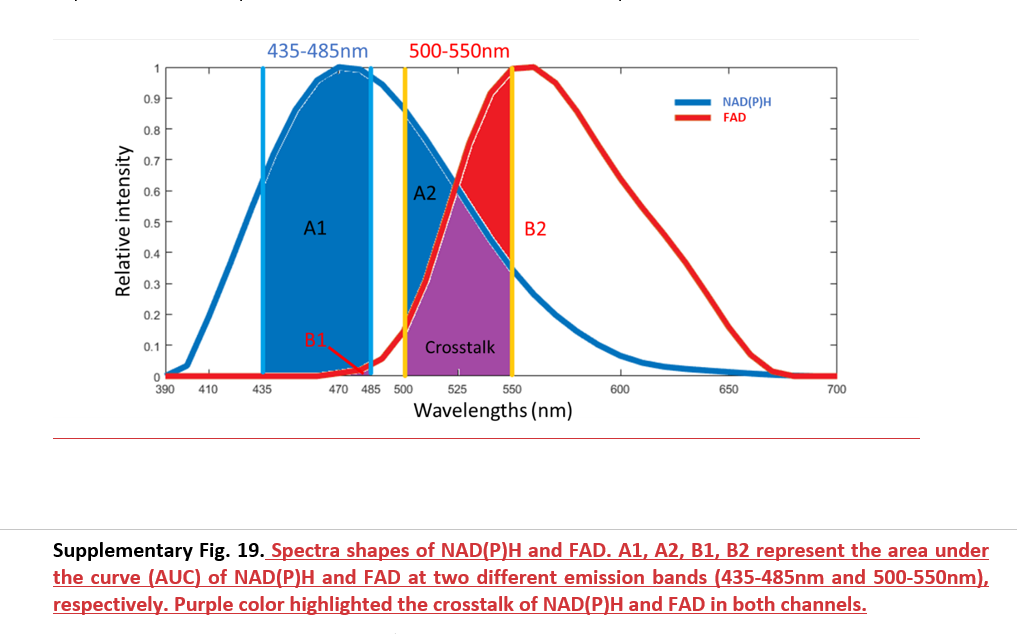
**

**Supplementary Fig. 34.** Spectra shapes of NAD(P)H and FAD. A1, A2. B1. B2 represents the area under the curve (AUC) of NAD(P)H and FAD at two different emission bands (435-485nm and 500-550nm), respectively. The purple color highlighted the crosstalk of NAD(P)H and FAD in both channels.

**
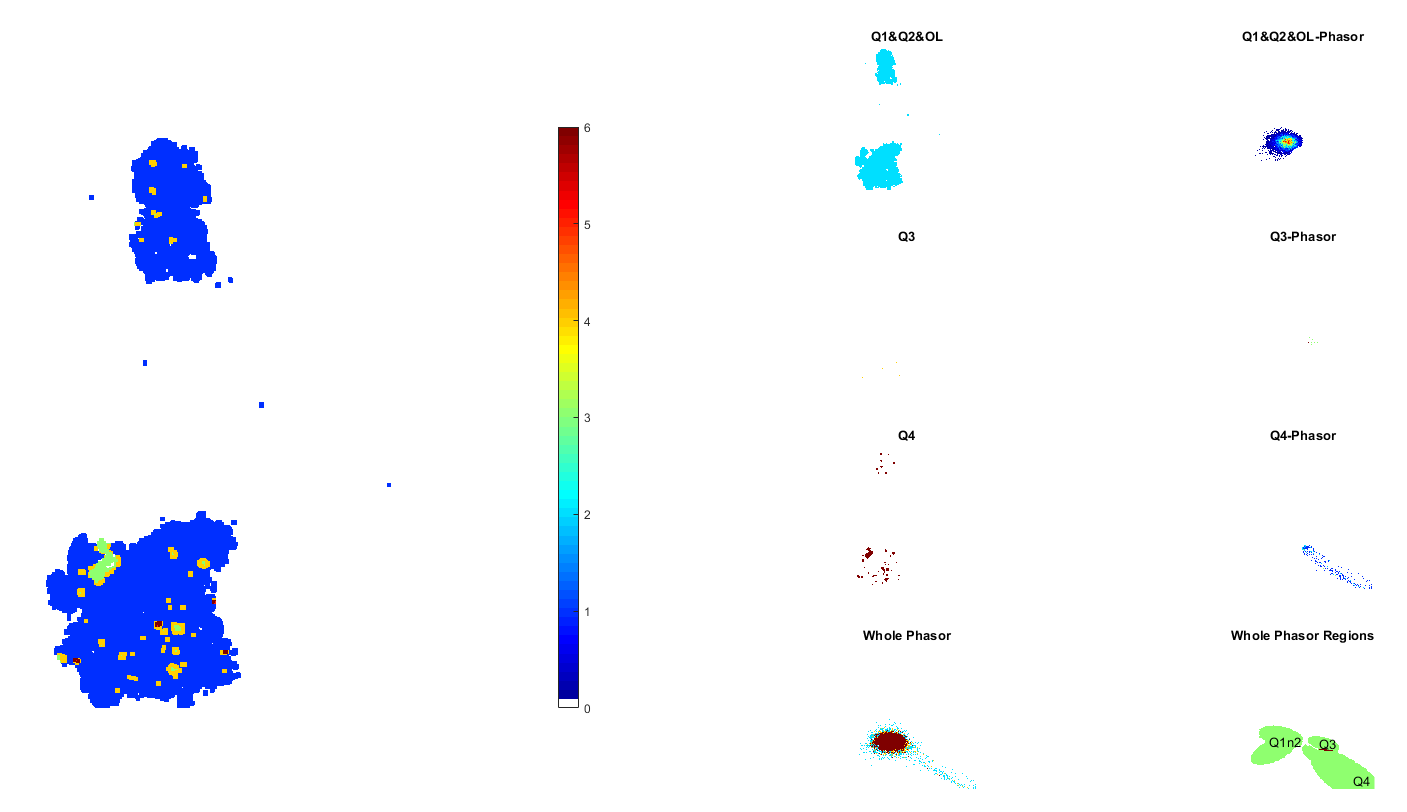
**

**Supplementary Fig. 35.** Representative Phasor-guided redox ratio area used for analysis.

**
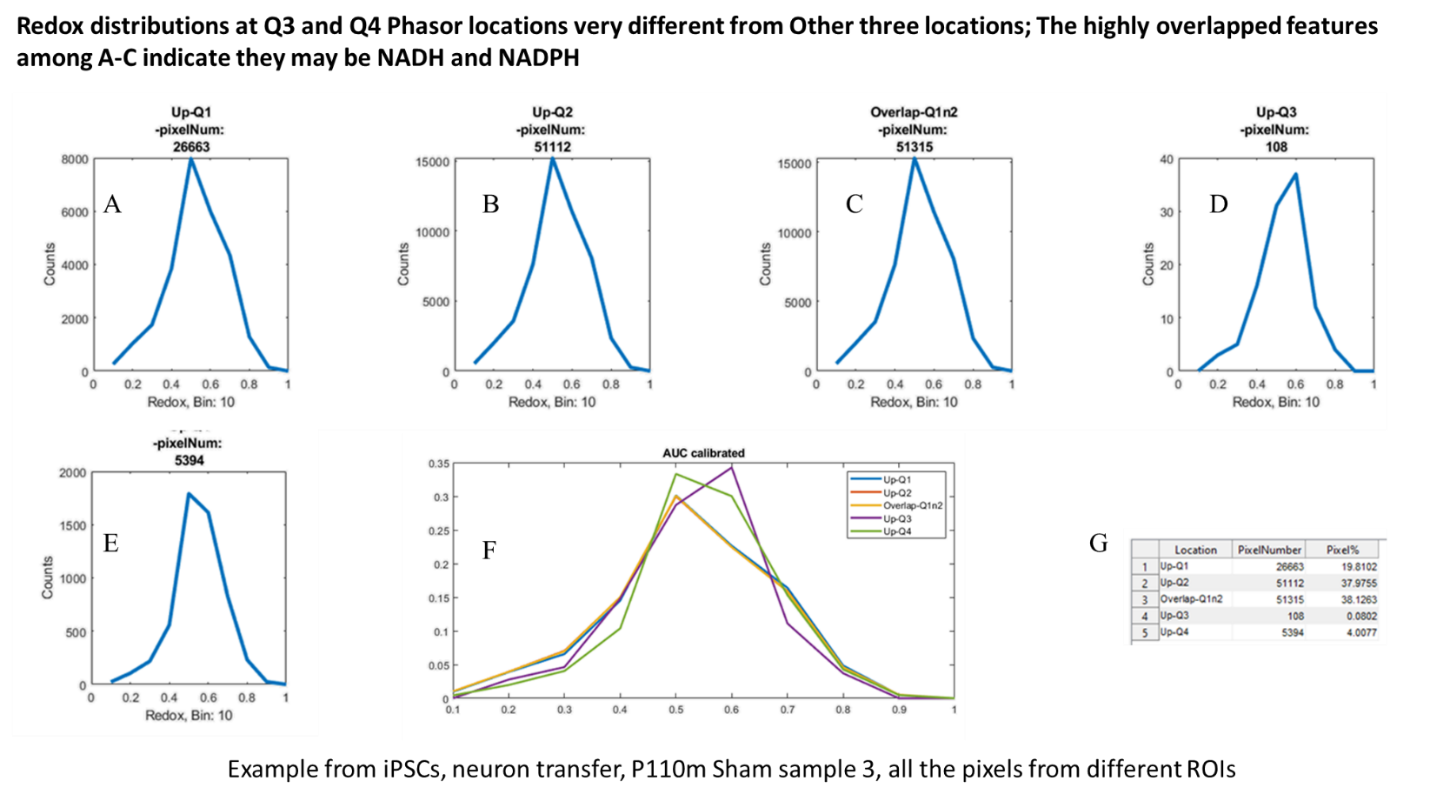
**

**Supplementary Fig. 36.** Redox distribution for each phasor location (A, B, D, E) and overlapped region between Q1 and Q2(C). Super-imposed AUC calibrated redox distribution from every different phasor location (F). (G) the pixel number percentage of different phasor locations.

1. Todd BP, Chimenti MS, Luo Z, Ferguson PJ, Bassuk AG, Newell EA. Traumatic brain injury results in unique microglial and astrocyte transcriptomes enriched for type I interferon response. J Neuroinflammation. 2021;18(1):151. Epub 2021/07/07. doi: 10.1186/s12974-021-02197-w. PubMed PMID: 34225752; PMCID: PMC8259035.

2. Burke EE, Chenoweth JG, Shin JH, Collado-Torres L, Kim SK, Micali N, Wang Y, Colantuoni C, Straub RE, Hoeppner DJ, Chen HY, Sellers A, Shibbani K, Hamersky GR, Diaz Bustamante M, Phan BN, Ulrich WS, Valencia C, Jaishankar A, Price AJ, Rajpurohit A, Semick SA, Burli RW, Barrow JC, Hiler DJ, Page SC, Martinowich K, Hyde TM, Kleinman JE, Berman KF, Apud JA, Cross AJ, Brandon NJ, Weinberger DR, Maher BJ, McKay RDG, Jaffe AE. Dissecting transcriptomic signatures of neuronal differentiation and maturation using iPSCs. Nat Commun. 2020;11(1):462. Epub 2020/01/25. doi: 10.1038/s41467-019-14266-z. PubMed PMID: 31974374; PMCID: PMC6978526.

3. Whalen MJ, Dalkara T, You Z, Qiu J, Bermpohl D, Mehta N, Suter B, Bhide PG, Lo EH, Ericsson M, Moskowitz MA. Acute plasmalemma permeability and protracted clearance of injured cells after controlled cortical impact in mice. J Cereb Blood Flow Metab. 2008;28(3):490-505. Epub 2007/08/24. doi: 10.1038/sj.jcbfm.9600544. PubMed PMID: 17713463; PMCID: PMC2711980.

4. Hinzman JM, Wilson JA, Mazzeo AT, Bullock MR, Hartings JA. Excitotoxicity and Metabolic Crisis Are Associated with Spreading Depolarizations in Severe Traumatic Brain Injury Patients. J Neurotrauma. 2016;33(19):1775-83. Epub 2015/11/21. doi: 10.1089/neu.2015.4226. PubMed PMID: 26586606; PMCID: PMC5065029.

5. Wu L, Chung JY, Cao T, Jin G, Edmiston WJ, 3rd, Hickman S, Levy ES, Whalen JA, Abrams ESL, Degterev A, Lo EH, Tozzi L, Kaplan DL, El Khoury J, Whalen MJ. Genetic inhibition of RIPK3 ameliorates functional outcome in controlled cortical impact independent of necroptosis. Cell Death Dis. 2021;12(11):1064. Epub 2021/11/11. doi: 10.1038/s41419-021-04333-z. PubMed PMID: 34753914; PMCID: PMC8578385.
