# Supplementary figures and images for: Mitochondria dysregulation contributes to secondary neurodegeneration progression post-contusion injury in human 3D in vitro triculture brain tissue model
Source: Cell Death Dis. 2023 Aug 3;14(8):496. doi: 10.1038/s41419-023-05980-0 (PMC10400598; doi:10.1038/s41419-023-05980-0)

Western Blot original files

pDRP1 over DRP1


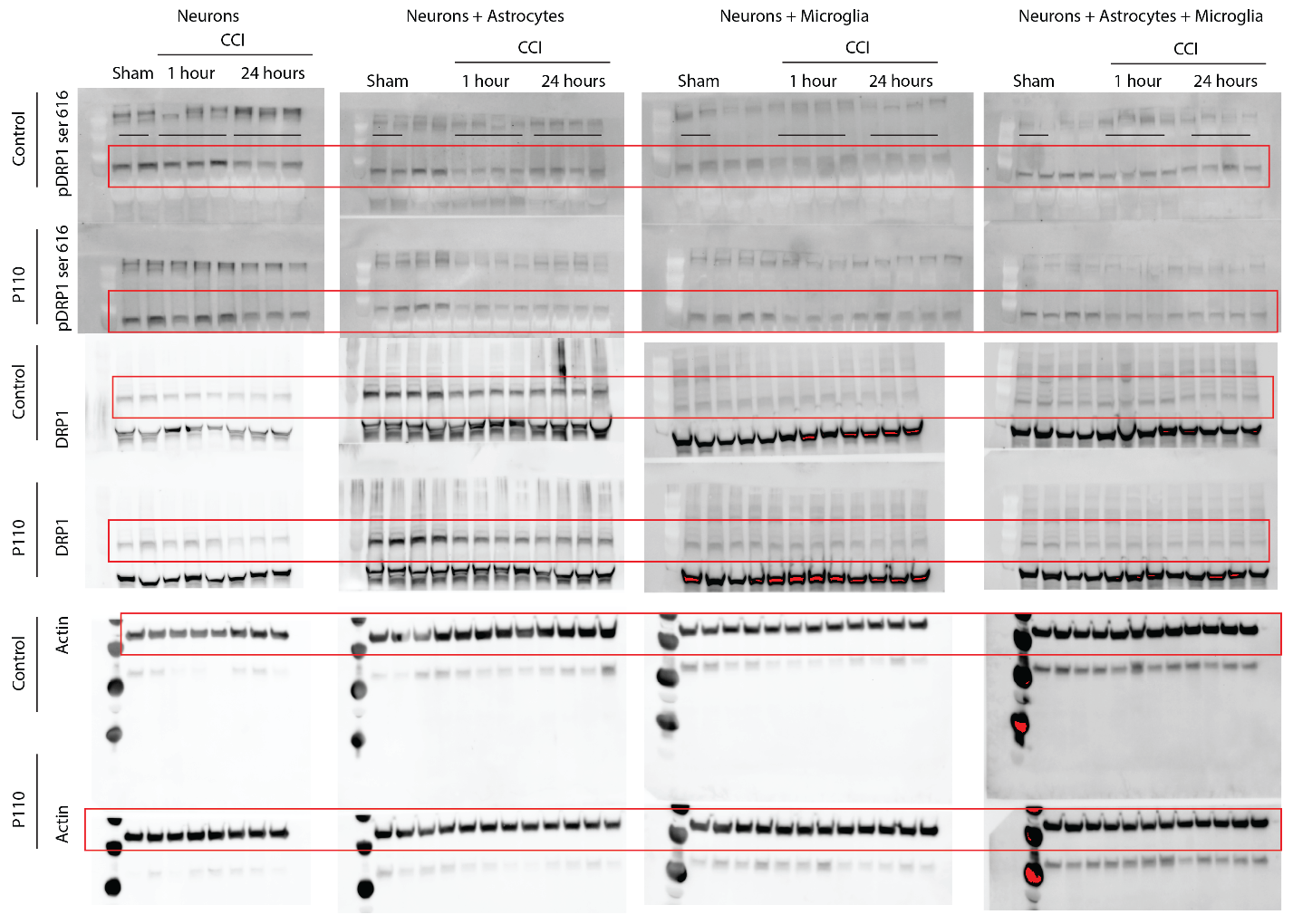


FIS1


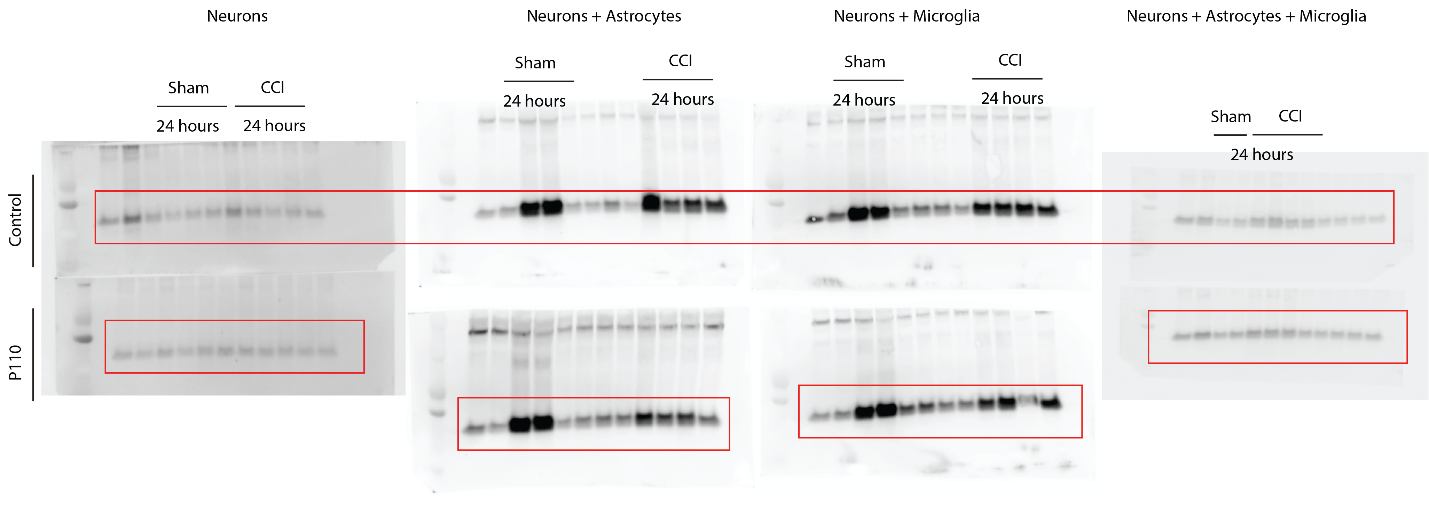


TOMM20


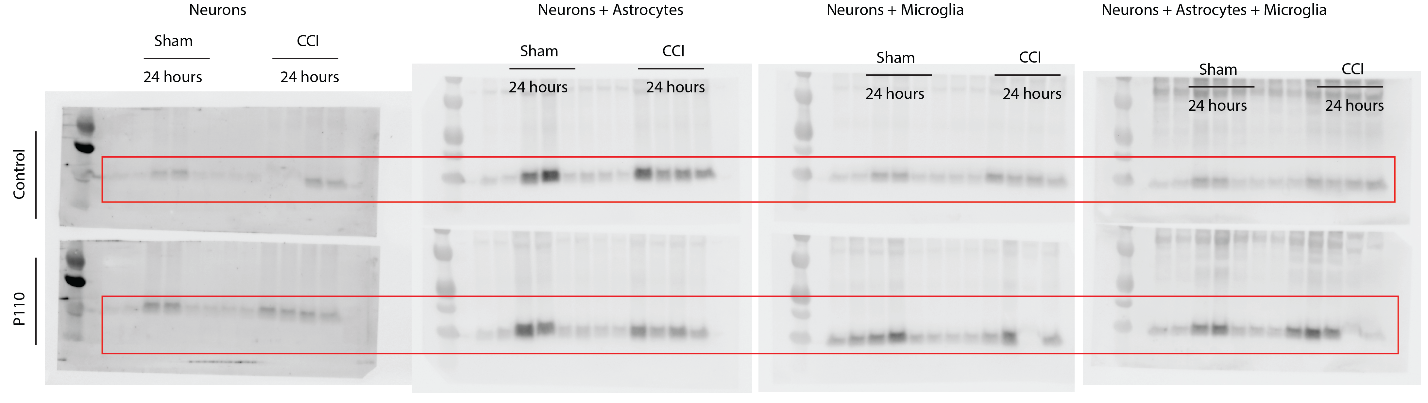


MAP2


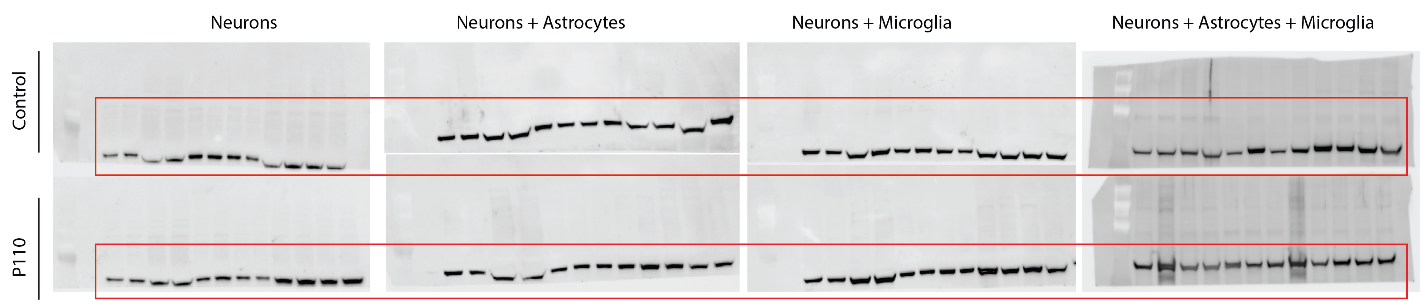

Supplement: Supplementary file 2 — ‘Original Data File’ [file 41419_2023_5980_MOESM2_ESM.docx]
